# Breakfast in the Philippines: food and diet quality as analyzed from the 2018 Expanded National Nutrition Survey

**DOI:** 10.1186/s12937-022-00804-x

**Published:** 2022-08-12

**Authors:** Imelda Angeles-Agdeppa, Ma. Rosel S. Custodio, Marvin B. Toledo

**Affiliations:** Department of Science and Technology, Food and Nutrition Research Institute, Bicutan, Taguig, Philippines

**Keywords:** Breakfast, Diet quality, Nutrient intake, NRF 9.3, Philippines

## Abstract

**Background:**

The quality of foods taken during breakfast could contribute in shaping diet quality. This study determined the regularity of breakfast consumption and breakfast quality based on the food, energy and nutrient intakes of Filipinos.

**Materials and methods:**

Data from the 2018 Expanded National Nutrition Survey (ENNS) was extracted for analysis. There were 63,655 individuals comprising about 14,013 school-aged children (6–12 years old), 9,082 adolescents (13–18 years old), 32,255 adults (19–59 years old), and 8,305 elderly (60 years old and above). Two-day non-consecutive 24-h food recalls were used to measure food and nutrient intakes. Diet quality was measured using Nutrient-Rich Food Index (NRF) 9.3. The sample was stratified by age group and NRF9.3 tertiles.

**Results and findings:**

Results showed that 96 – 98% Filipinos across age groups were consuming breakfast. Children age 6–12 years have the highest NRF9.3 average score (417), followed by the elderly (347), adolescents (340), and adults (330). These scores were very low in comparison with the maximum possible NRF score which is 900. The essential nutrient intakes of respondents were significantly higher among those with the healthiest breakfast diet (Tertile 3) compared to those with the poorest breakfast diet (Tertile 1). However, participants in the healthiest breakfast diet did not meet 20% of the recommendations for calcium, fiber, vitamin C, and potassium.

**Conclusion and recommendations:**

This study revealed that majority of the population are regular breakfast consumers. However, the breakfast consumed regularly by Filipinos were found to be nutritionally inadequate. And even those classified under Tertile 3 which were assumed as having a better quality of breakfast were still found to have nutrient inadequacies. Thus, the study suggests that Filipinos must consume a healthy breakfast by including nutrient-dense foods such as fruits, vegetables, whole grains, fresh meat, and milk to provide at least 20–25% of the daily energy and nutrient intakes.

**Supplementary Information:**

The online version contains supplementary material available at 10.1186/s12937-022-00804-x.

## Introduction

Breakfast is often regarded as the most important meal of the day. The American Heart Association defines breakfast as the first meal of the day eaten within 2 h after waking up [1]. Latest evidences from previous studies suggest consuming about 15 – 25% of the daily energy intake at breakfast [2], wherein the composition of the foods consumed should be from the five main food groups such as: Starchy foods, fruits and vegetables, milk and dairy, protein sources and low-fat spreads and oils. Other recommendations from nutrition and dietetics institutes suggest that breakfast consumption is a key component to an optimal diet which improves cognitive function and helps control against weight gain [3, 4]. It was also found that a breakfast high in protein and fat proves to aid in the management of glycemic index among type 2 diabetics [5]. The International Breakfast Research Initiative (IBRI) aimed to develop nutritional guidelines for a healthy breakfast by conducting a standardized study of national nutrition surveys from selected Asian countries [6].

Throughout the years, a significant amount of literature has supported the beneficial effects of breakfast consumption. Several studies have consistently recorded a broad variance in breakfast’s contribution to nutrient intake and diet quality in different parts of the world [7–9]. Daily breakfast intake was correlated with higher intakes of healthier foods such as whole grains, dairy, and vegetables, thus giving breakfast consumers a higher tendency to achieve recommended nutrient intakes [10]. In adolescents, there is a reduced risk of becoming overweight or obese [12,13]. In terms of mental health, it was found out that breakfast consumers were less likely to be emotionally distressed, have lower risk of depression and perceived stress compared to its opposite counterpart [14].

In the Philippines, there is limited information about breakfast consumption.

A typical Filipino diet in a day consists of about three and a half (3 ½) cups of cooked rice, one (1) matchbox of fried fish, and half (1/2) cup of boiled vegetables per day and these are usually consumed during the three (3) major meals of the day: breakfast, lunch and supper []. Previous studies found that breakfast food pattern in the Philippines and specific provinces was rice, bread, fish, egg, and coffee which were mainly composed of foods rich in carbohydrates, protein, and fat [16,17]. It was noticeable that the regular breakfast of Filipinos was lacking in vitamins and minerals which are commonly found in fruits and vegetables. In a study with 45 severely wasted children, the respondents “always” eat their lunch and dinner at home and “sometimes” only for breakfast and after the school feeding program was implemented, the respondents' nutritional status significantly improved to normal [18]. Research has pointed out that nutrients that are unattained during breakfast are not compensated by meals in the later day, thus stressing the importance of breakfast consumption in meeting daily nutrient requirement [19]. The overarching objective of this study determined the regularity of breakfast consumption and breakfast quality based on the food, energy and nutrient intakes of Filipinos.

## Methodology

### Study population

**Fig. 1:**
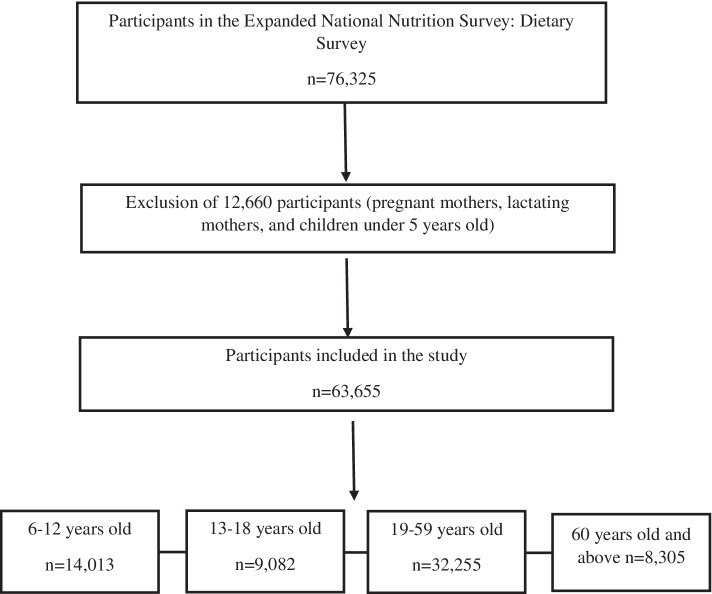
Flowchart for sample selection. Data from the 2018 ENNS was used with a starting sample size of 76,325. After exclusion of participants and checking of completeness of the data, final participants included in the study is 63,655

For this study, data was derived from the 2018 Expanded National Nutrition Survey (ENNS) dietary component survey. The coverage population of the ENNS is about 40 provinces with over 80,540 surveyed individuals with a response rate of 81.5%. The 2018 ENNS utilized the 2013 Master Sample List developed by the Philippine Statistics Authority (PSA). This sampling design followed a two-stage cluster sampling technique. The first stage involved the selection of Primary Sampling Units (PSUs), which involved sampling domains from 81 provinces, wherein 16 sample replicates was drawn from each domain. The second stage involved the selection of households from the 16 sample replicates. During this stage, the selected households served as the final sampling unit [20]. The final sample size that is included in this study was *n* = 63,655. The breakdown per age of the total sample size are as follows: 14,013 school-aged children (6–12 years old), 9,082 Adolescents (13–18 years old), 32,255 Adults (19–59 years old), and 8,305 Elderly (60 years old and above) Fig. 1.

### Breakfast regularity and dietary data

Breakfast was defined based on the meal code number 1 indicated in the questionnaire which was self-reported as “Breakfast” by the interviewee. On the other hand, breakfast skipping was defined in this study as no breakfast consumption or less than 50 kcals of energy consumed at breakfast otherwise regular consumer if more than or had 50 kcal [10]. Two day non-consecutive 24-h dietary recalls were done face-to-face with each participant or child's parent or caregiver. The interviews were conducted by trained registered dietitians during home visits using a prepared questionnaire. The initial 24 h dietary recall was collected for all respondents, and a second 24 h dietary recall was completed in 50% of randomly selected households only on a non-consecutive day to estimate the day-to-day variation component in energy and nutrient consumption necessary for habitual intake analysis. The second 24 h dietary recall was generally obtained 2 days after the first 24 h recall. Food records were encoded and estimated energy and nutrient intakes were processed using the electronic–based Individual Dietary Evaluation System (IDES) developed by the Institute. This system contains the data of the updated Filipino Food Composition Tables (FCT) [21].

### NRF9.3 scoring of breakfast consumption

Diet quality was measured using the Nutrient Rich Food Index (NRF) 9.3. The NRF9.3 is a validated measurement tool to determine the nutrient density of the total diet. The determination of the nutrient density score for the NRF 9.3 is calculated by the sum of the percentage daily reference values (DRVs) of the nine nutrients that are recommended (protein, dietary fiber, vitamin A, C, and D, calcium, iron, potassium, and magnesium) minus the sum of the percentage of DRVs for the three nutrients to limit (added sugar, saturated fat, and sodium). Total sugar intake was used since added sugar was not included in the survey. The closer the value is to the maximum score of 900 would indicate better quality of the overall diet. The basis for the NRF9.3 algorithm was from a nutrient profiling model on linking food items that have the highest nutrient density at the same time, affordable and engaging [20, 22, 23].

The Philippine Dietary Reference Intake (PDRI) and other standard recommendations were used to calculate the reference daily values (DVs) []. The following were the qualifying nutrients and standard reference amounts: protein (50 g), fiber (25 g), vitamin A (1500 RE), vitamin C (60 mg), vitamin D (10 mcg), calcium (1000 mg), iron (18 mg), potassium (3500 mg) and magnesium (400 mg). The 3 disqualifying nutrients and maximum recommended values (MRVs) were: added sugar (50 g), saturated fat (20 g) and sodium (2400 mg).

The following is the NRF 9.3 calculation formula:

$$NRF9.3=\left(NR-LIM\right) \times 100$$

with

$$NR= \sum_{i=1}^{9}\frac{{Intake}_{i}/ED\times 2000}{{DV}_{i}}$$

and

$$LIM={\textstyle\sum_{i=1}^3}\frac{{Intake}_i/ED\times2000}{{MRV}_i}-1$$

where *intake*_i_ is the intake of each nutrient, ED is the energy density, and *DV*_*i*_ is the reference daily value for that nutrient. In NR calculation, the intake of each nutrient for each subject was normalized for 2000 kcal and expressed as a percentage of the reference DVs. Previously, nutritional % DVs were capped at 100, so that an extremely high intake of one nutrient could not compensate for a dietary deficiency of another. Only the portion in excess of the recommended quantity was taken into account for the LIM.

### Commonly consumed food at breakfast

Food group consumption was express as actual intake (in grams) and percentage of consumer. Consumers of each food group were scored 1 if they consumed at least 10 g otherwise 0 (< 10 g) [27]. Only the top 10 mostly consumed food groups were presented in this study.

### Contribution of breakfast to the daily intake and recommendation

The percentage contribution of breakfast to daily nutrient intake was calculated by calculating the total intake of each nutrient at breakfast divided by the total intake of each nutrient at daily intake multiplied by 100. The percentage contribution of breakfast among people with the healthiest breakfast quality to the daily recommendation was calculated by dividing their intake to the recommendation for each nutrient and their respective recommendations and then multiplied by 100. Same calculation was done for the percentage contribution of nutrient intake from all meals to daily recommendation.

### Outlier determination

Quality control of the dietary intake data was conducted in two steps. In the first step, the foods reported by a participant like coding information, and quantity were reviewed. In the second step, scatterplots and histograms was used to determine the outliers of the datasets. For implausible micronutrient intake, excessive intakes were defined as those that exceeded 1.5 times of the 99th percentile of the observed intake distribution of the nutrient in the corresponding sex, and age group. Intakes above the upper limit were substituted by a random value generated from a uniform distribution in the intervals with lower bound equal to the 95th percentile of the observed intake and an upper bound equal to 1.5 times the 99th percentile [28]. After validation and checking for completeness, a total of 12,670 participants were excluded in this study. This included pregnant mothers, lactating mothers, children 5 years old and below, and outliers.2.3. Measurement of Diet Quality.

### Statistical analysis

PCSIDE version 1.02 (Iowa State University, Ames, IA, USA) was utilized in estimating the usual nutrient intakes at breakfast. This program estimates distributions of usual nutrient intake by removing the effect of day-to-day (intra-person) variability in intake from daily intakes [29, 30]. All statistical analyses were performed in the STATA version 15 (StataCorp, USA) software. Analysis of variance (ANOVA) was used to test the mean difference of NRF 9.3 scores, energy and nutrients intakes between age groups. Differences in food group consumption were tested using Analysis of Covariance (ANCOVA), adjusted for energy at breakfast as well as sociodemographic characteristics. NRF9.3 score were group into tertiles using –xtile- command in STATA, where, Tertile 1 represent the group with the poorest breakfast quality and Tertile 3 have the healthiest breakfast quality. A trend test was conducted to test whether nutrient intakes at breakfast tends to either increase or decrease across NFR tertiles. Chi-square test was conducted to test the association between consumption of food group and NRF 9.3 Score in tertiles. To achieve estimates at a population level, weighting recommendations was followed in all analyses using “svy” command. The significance level was set at *p* < 0.05.

## Results

### Breakfast regularity across participants’ characteristics

Table 1 shows that 96–98% are breakfast consumers. Only 2% of the younger age groups (6–12 yo and 13–18 yo) were breakfast skippers while for both adults and elderly groups, the percentage slightly increased to 4%. Almost all participants were breakfast consumers across demographic characteristics and nutritional statuses (male 95–98%, female 96–98%). There was a significant association between breakfast consumption and gender among adults only. Proportion of female breakfast consumers (96%) was higher compared to males (95%). In contrast to urban areas, higher percentage of breakfast skippers were significantly observed in rural areas among adolescents (2.3%), adults (4.7%) and elderly (4.8%) groups. It appears that proportions of breakfast consumers across wealth quintile was almost equivalent for all age groups. Educational attainment was significantly associated to breakfast consumption among adults and elderly group. For adults, the proportion of breakfast consumers who have an elementary and college level education was 94% and 97%, respectively. On the other hand, the proportion of breakfast consumers among the elderly with elementary level and college level education was 95% and 98%, respectively. Nutritional status of the children and adolescents was not associated to breakfast status. Elderly with chronic energy deficiency (CED) (94%) has the lowest proportion of breakfast consumers (94%), while overweight (97%) and obese (98%) elderly has the highest proportion of breakfast consumers.

**Table 1:** Breakfast regularity across demographics and nutritional status

		**6–12 Years Old**		**13–18 Years Old**		**19–59 Years Old**		**60 Years Old and Above**	
		**Skippers**	**Consumers**	**Skippers**	**Consumers**	**Skippers**	**Consumers**	**Skippers**	**Consumers**
	**Sample, n**	**N (%)**	**N (%)**	**N (%)**	**N (%)**	**N (%)**	**N (%)**	**N (%)**	**N (%)**
**All**	63, 655	212 (1.7)	13, 801 (98.3)	166(1.8)	8, 916 (98.2)	1,164 (4)	31, 091 (96)	316 (4)	7, 989 (96)
**Sex**									
Male	30, 722	99 (1.5)	7,072 (98.5)	87 (2)	4,526 (98)	600 (4.5)	14, 886 (95.5)	148 (4.3)	3,304 (95.7)
Female	32, 933	113 (1.9)	6,729 (98.1)	79 (1.7)	4,390 (98.3)	564 (3.6)	16, 205 (96.4)	168 (3.8)	4,685 (96.2)
*p-value*^*1*^		*0.270*		*0.331*		0.019*		*0.413*	
**Urbanity**									
Rural	42, 459	159 (1.9)	9,391 (98)	138 (2.3)	6,190 (97.7)	834 (4.7)	20, 105 (95.3)	235 (4.8)	5,407 (95.2)
Urban	21, 196	53 (1.3)	4,410 (98.7)	28 (1.1)	2,726 (98.9)	330 (3.2)	10, 986 (96.8)	81 (3)	2,582 (97)
*p-value*^*1*^		*0.302*		0.003*		0.045*		0.002*	
**Wealth Quintile**									
Poorest	16, 251	93 (2.3)	4,243 (97.7)	51 (2.3)	2,319 (97.7)	308 (4.3)	7, 394 (96.7)	71 (3.9)	1,772 (96)
Poor	15, 489	61 (2)	3,519 (98)	52 (2.4)	2,332 (97.6)	296 (4.4)	7, 342 (95.6)	89 (5.7)	1,798 (94.3)
Middle	12, 603	29 (1.5)	2,605 (98.4)	33 (1.7)	1,802 (98.3)	233 (4.1)	6, 313 (95.8)	62 (3.9)	1,526 (96.1)
Rich	10, 288	16 (0.9)	1,960 (99.1)	24 (1.6)	1,360 (98.4)	153 (3.4)	5, 364 (96.6)	47 (3.5)	1,364 (96.4)
Richest	8, 700	10 (0.9)	1,390 (99.1)	6 (0.8)	1,052 (99.2)	158 (3.6)	4, 532 (96.4)	46 (3.1)	1,506 (96.9)
*p-value*^*1*^		*0.325*		*0.245*		*0.169*		*0.112*	
**Education**									
Elementary Level	28, 922	-	-	-	-	409 (5.4)	8,429 (94.5)	209 (5)	4,424 (95)
High School Level	22, 545	-	-	-	-	458 (3.9)	12,607 (96.1)	76 (3.8)	1,974 (96.2)
College Level	9, 510	-	-	-	-	217 (2.9)	7,846 (97.1)	25 (1.7)	1,323 (98.3)
Vocational Level	2, 676	-	-	-	-	80 (3.7)	2,207 (96.3)	6 (2.2)	268 (97.8)
*p-value*^*1*^		*-*		*-*		< 0.001*		0.019*	
**WAZ Status (only for 6–10 years old)**									
Severely Underweight	488	13 (2.8)	475 (97.2)	-	-	-	-	-	-
Underweight	1,772	37 (2.2)	1,735 (97.8)	-	-	-	-	-	-
Normal	5, 982	84 (1.6)	5,898 (98.5)	-	-	-	-	-	-
Above Normal	227	2 (1.4)	225 (98.5)	-	-	-	-	-	-
*p-value*^*1*^		*0.359*							
**HAZ Status (only for 6–19 years old)**									
Severely Stunted	1, 213	17 (2.6)	676 (97.4)	11 (1.4)	499 (98.5)	-	-	-	-
Stunted	5, 117	55 (1.9)	2,849 (98.1)	31 (1.3)	2,151 (98.7)	-	-	-	-
Normal	16, 585	139 (1.6)	10,116 (98.4)	122 (2)	6,150 (97.9)	-	-	-	-
Tall	51	0	0	0	1 (100)	-	-	-	-
*p-value*^*1*^		*0.520*		0.134					
**BAZ Status (only for 6–19 years old)**									
Severely Thin	423	2 (0.6)	232 (99.4)	3 (1)	184 (99)	-	-	-	-
Thin	2, 009	19 (1.6)	1,149 (98.4)	19 (2.4)	811 (97.5)	-	-	-	-
Normal	18, 318	183 (1.8)	10,858 (98.1)	132 (1.8)	7,068 (98.2)	-	-	-	-
Overweight	1, 438	5 (0.7)	893 (99.3)	5 (1.1)	529 (98.8)	-	-	-	-
Obese	778	2 (0.6)	559 (99.4)	5 (2.9)	209 (97)	-	-	-	-
*p-value*^*1*^		*0.261*		0.725					
**BMI Status (only for adults)**									
Chronic energy Deficiency (CED)	3, 253	-	-	-	-	90 (4.6)	2,038 (95.4)	58 (5.7)	1,067 (94.3)
Normal	21, 418	-	-	-	-	654 (4.2)	16,446 (95.7)	180 (4.3)	4,138 (95.6)
Overweight	10, 409	-	-	-	-	291 (3.7)	8,320 (96.3)	47 (3.2)	1,751 (96.7)
Obese	3, 065	-	-	-	-	83 (3.4)	2,534 (96.6)	10 (1.7)	438 (98.3)
*p-value*^*1*^						*0.168*		0.006*	

*WAZ* stand for Weight-for-Age zscore, *HAZ* for Height-for-Age zscore, *BAZ* for BMI-for-Age zscore, *BMI* stands for Body Mass Index

^1^Chi-square test for association with α = 0.05, *significant

### Consumption of commonly consumed food at breakfast by NRF9.3 tertiles

Tables 2, 3, 4 and 5 presents the average consumption of the top 10 mostly consumed food groups. The NFR 9.3 tertiles are also shown for the age groups including the percentage consumption of each food group. The analyses showed that consumption of all top 10 food groups were associated to NRF tertiles. The highlight of this result explains that individuals with the Tertile 3 consumed more vegetables, fresh meat and fish, and eggs but less in rice, sugar and coffee.

In Table 2, children with poorest quality diets (Tertile 1) have a higher percentage consumption of cereal products (51%) and sugars (27%) while having lower consumption of powdered milk (11%), other vegetables (2%), green leafy vegetables (0.4%), fresh fish (2%), chicken eggs (6%), cacao and chocolate-based beverages (1%), and rice (49%). For adolescent group in Table 3, results show that Tertile 1 has a higher percentage consumption of coffee (57%), sugars (30%), and cereals products (52%) while having the lowest percentage consumption of green leafy vegetables (0.4%), other vegetables (3%), cacao and chocolate-based beverages (0.3%), chicken egg (6%), fresh fish (2%), and rice (43%) compared to T2 and T3. Consumption of coffee seems to increase as age increases with tertile 1 having the highest consumption including sugars. This is evident in the analysis for the adults and elderly group. In Table 4, results showed that adults aged 19–59 years old in Tertile 1 has the highest percentage consumption of coffee (85%), and cereal products (47%), while having the lowest percentage consumption of green leafy vegetables (0.4%), other vegetables (2%), and chicken egg (7%), fresh fish (2%), fresh meat (1%) and rice (29%) compared to T2 and T3. On the other hand, results shown in Table 5 for the elderly group aged 60 years old and above shows that Tertile 1 has the highest percentage consumption of coffee (88%), and cereal products (48%) while having the lowest percentage consumption of green leafy vegetables (0.4%), other vegetables (2%), powdered milk (3%), chicken egg (8%), fresh fish (3%), and rice (29%) compared to T2 and T3. For all age groups, Tertile 3 has the highest mean consumption of fresh fish, green leafy vegetables and other vegetables while having a moderate consumption of rice, chicken egg, and cooking oil. Younger age groups such as school age children and adolescents who belong to T3 had the highest consumption of chocolate-based beverages. While older adults and elderly in T3 had the highest consumption of rice and the least consumption of coffee.

**Table 2:** Mean food group intake and percentage consumers at breakfast for Children by NRF 9.3 Tertiles, Philippines, 2018

**Food Groups**	**6–12 years old (*n* = 13, 801)**							
	**Mean Food Group Intake (g) **^a^			***P*-value**†	**Percentage Consumer (%)**^b^			***P*-value**^‡^
	**Tertile 1**	**Tertile 2**	**Tertile 3**		**Tertile 1**	**Tertile 2**	**Tertiles 3**	
RICE	65.1 ± 1.27	68.5 ± 0.85	58 ± 0.81	< 0.001^abc^	49 ± 0.01	80 ± 0.01	76 ± 0.01	< 0.001*
COOKING OIL	4.8 ± 0.18	4.3 ± 0.09	4.1 ± 0.08	0.335	26 ± 0.01	49 ± 0.01	31 ± 0.01	< 0.001*
CACAO AND CHOCOLATE BASED BEVERAGES	29.9 ± 8.69	24.4 ± 4.13	21.4 ± 0.60	0.065	1 ± 0.001	3 ± 0.003	27 ± 0.01	< 0.001*
CHICKEN EGG	24.1 ± 1.19	34.4 ± 0.63	40 ± 0.73	< 0.001^abc^	6 ± 0.003	31 ± 0.01	26 ± 0.01	< 0.001*
OTHER CEREAL PRODUCTS	51.1 ± 1.03	53.8 ± 1.57	44.9 ± 1.40	< 0.001^bc^	51 ± 0.01	24 ± 0.01	19 ± 0.01	< 0.001*
FRESH FISH	28.7 ± 1.76	35.5 ± 0.94	41.1 ± 1.42	< 0.001^ab^	2 ± 0.002	16 ± 0.005	17 ± 0.005	< 0.001*
SUGARS	7.5 ± 0.20	6.7 ± 0.24	6.4 ± 0.25	< 0.001^ab^	27 ± 0.01	21 ± 0.01	15 ± 0.005	< 0.001*
GREEN LEAFY VEGETABLES	10 ± 2.20	14.5 ± 1.79	22.8 ± 1.19	< 0.001^b^	0.3 ± 0.001	2 ± 0.002	14 ± 0.005	< 0.001*
OTHER VEGETABLES	25.4 ± 2.20	28.6 ± 2.19	39 ± 2.66	< 0.001^b^	2 ± 0.002	8 ± 0.004	13 ± 0.005	< 0.001*
POWDERED MILK	24.6 ± 0.62	22.6 ± 0.83	21.6 ± 0.63	< 0.001^ab^	11 ± 0.005	24 ± 0.01	13 ± 0.005	0.007*

^a^Values are mean ± standard error

^b^Values are percentage ± standard error

^†^Using Analysis of Covariance adjusted for age, sex, urbanity, wealth quintiles and energy intake at breakfast using Turkey HDS posthoc pairwise comparisons; subscripts represents significant difference between ^a^Tertile 1 vs Tertile 2, ^b^Tertile 1 vs Tertile 3, and ^c^Tertile 2 vs Tertile 3

^‡^Using Rank Biserial Correlation test; ^*^significant

**Table 3:** Mean food intake and percentage consumer at breakfast for Adolescents by NRF 9.3 Tertiles, Philippines, 2018

**Food Groups**	**13–18 years old (*n* = 8, 916)**							
	**Mean Food Group Intake (g)**^a^			**Percentage Consumer (%)**^b^				
	Tertile 1	Tertile 2	Tertile 3	*P*-value^†^	Tertile 1	Tertile 2	Tertile 3	*P*-value^‡^
RICE	95.5 ± 1.77	111.8 ± 1.63	93.8 ± 1.40	< 0.001^abc^	43 ± 0.01	81 ± 0.01	77 ± 0.01	< 0.001*
COOKING OIL	6.7 ± 0.32	5 ± 0.15	4.7 ± 0.14	< 0.001^ab^	22 ± 0.01	49 ± 0.01	32 ± 0.01	< 0.001*
FRESH FISH	34.6 ± 5.26	47.5 ± 2.40	65.1 ± 3.73	< 0.001^abc^	2 ± 0.003	16 ± 0.01	22 ± 0.01	< 0.001*
CHICKEN EGG	30.5 ± 2.32	38 ± 0.88	46.5 ± 1.51	< 0.001^abc^	6 ± 0.004	30 ± 0.01	22 ± 0.01	< 0.001*
OTHER CEREAL PRODUCTS	59.9 ± 1.56	62.2 ± 2.42	65.2 ± 3.97	< 0.001^abc^	52 ± 0.01	19 ± 0.01	17 ± 0.01	< 0.001*
CACAO AND CHOCOLATE BASED BEVERAGES	12.9 ± 2.25	21.1 ± 1.59	20.7 ± 0.44	0.019^ab^	0.4 ± 0.001	2 ± 0.002	17 ± 0.01	< 0.001*
OTHER VEGETABLES	29.3 ± 4.51	39.5 ± 2.31	56.5 ± 3.21	< 0.001^bc^	3 ± 0.003	12 ± 0.01	16 ± 0.01	< 0.001*
SUGARS	9.4 ± 0.33	8.5 ± .36	6.9 ± 0.33	< 0.001^abc^	30 ± 0.01	21 ± 0.01	15 ± 0.01	< 0.001*
GREEN LEAFY VEGETABLES	11.3 ± 2.18	18.4 ± 2.03	33.1 ± 1.91	< 0.001^b^	0.5 ± 0.001	3 ± 0.003	15 ± 0.01	< 0.001*
COFFEE	16.2 ± 0.38	13.4 ± 0.58	10.4 ± 0.72	< 0.001^abc^	57 ± 0.01	26 ± 0.01	12 ± 0.01	< 0.001*

^a^Values are mean ± standard error

^b^Values are percentage ± standard error

^†^Using Analysis of Covariance adjusted for age, sex, urbanity, wealth quintiles and energy intake at breakfast using Turkey HDS posthoc pairwise comparisons; subscripts represents significant difference between ^a^Tertile 1 vs Tertile 2, ^b^Tertile 1 vs Tertile 3, and ^c^Tertile 2 vs Tertile 3

^‡^Using Rank Biserial Correlation test; ^*^significant

**Table 4:** Mean food intake and percentage consumer at breakfast for Adults by NRF 9.3 Tertiles, Philippines, 2018

**Food Groups**	**19–59 years old (*n* = 31, 091)**							
	**Mean Food Group Intake (g)**^a^			*P*-value^†^	**Percentage Consumer (%)**^b^			*P*-value^‡^
	Tertile 1	Tertile 2	Tertile 3		Tertile 1	Tertile 2	Tertile 3	
RICE	90.8 ± 1.19	113.6 ± 0.87	105 ± 0.82	< 0.001^abc^	29 ± 0.004	75 ± 0.004	78 ± 0.004	< 0.001*
COFFEE	18.8 ± 0.18	13.4 ± 0.21	10.6 ± 0.25	< 0.001^abc^	85 ± 0.004	62 ± 0.005	39 ± 0.004	< 0.001*
SUGARS	8.7 ± 0.14	8.3 ± 0.13	7.7 ± 0.13	< 0.001^abc^	32 ± 0.005	37 ± 0.005	30 ± 0.004	< 0.001*
COOKING OIL	6.9 ± 0.21	5.3 ± 0.09	5.1 ± 0.09	< 0.001^ab^	16 ± 0.004	43 ± 0.005	28 ± 0.004	< 0.001*
FRESH FISH	26.2 ± 1.60	48.3 ± 1.23	64.8 ± 1.14	< 0.001^abc^	2 ± 0.001	19 ± 0.004	27 ± 0.004	< 0.001*
OTHER VEGETABLES	23.1 ± 2.42	45.4 ± 1.62	62.2 ± 1.71	< 0.001^abc^	2 ± 0.001	13 ± 0.003	21 ± 0.004	< 0.001*
GREEN LEAFY VEGETABLES	6.9 ± 1.32	17.7 ± 0.99	36.8 ± 1.04	< 0.001^bc^	0.3 ± 0.001	3 ± 0.002	20 ± 0.004	< 0.001*
CHICKEN EGG	28.5 ± 0.70	39.2 ± 0.49	45.4 ± 0.79	< 0.001^abc^	7 ± 0.002	26 ± 0.004	18 ± 0.004	< 0.001*
OTHER CEREAL PRODUCTS	61.5 ± 0.94	63.5 ± 1.27	55.7 ± 1.49	< 0.001^bc^	47 ± 0.005	24 ± 0.004	15 ± 0.003	< 0.001*
FRESH MEAT	40.9 ± 3.86	53.5 ± 2.48	74.9 ± 2.26	< 0.001^bc^	1 ± 0.001	7 ± 0.002	11 ± 0.003	< 0.001*

^a^Values are mean ± standard error

^b^Values are percentage ± standard error

^†^Using Analysis of Covariance adjusted for age, sex, urbanity, wealth quintiles and energy intake at breakfast using Turkey HDS posthoc pairwise comparisons; subscripts represents significant difference between ^a^Tertile 1 vs Tertile 2, ^b^Tertile 1 vs Tertile 3, and ^c^Tertile 2 vs Tertile 3

^‡^Using Rank Biserial Correlation test; ^*^significant

**Table 5:** Mean food intake and percentage consumer at breakfast for Elderly by NRF 9.3 Tertiles, Philippines, 2018

**Food Groups**	**60 years old and above (*n* = 7, 989)**							
	**Mean Food Group Intake (g)**^a^			*P*-value^†^	**Percentage Consumer (%)**^b^			*P*-value^‡^
	Tertile 1	Tertile 2	Tertile 3		Tertile 1	Tertile 2	Tertile 3	
RICE	72.6 ± 1.78	89.5 ± 1.49	76 ± 1.24	< 0.001^ac^	29 ± 0.01	66 ± 0.01	74 ± 0.01	< 0.001*
COFFEE	16.3 ± 0.35	10.4 ± 0.35	8.9 ± 0.43	< 0.001^abc^	88 ± 0.01	65 ± 0.01	42 ± 0.01	< 0.001*
SUGARS	9 ± 0.33	7.7 ± 0.23	7.1 ± 0.22	< 0.001^ab^	40 ± 0.01	47 ± 0.01	35 ± 0.01	0.001*
FRESH FISH	29.7 ± 3.4	39.7 ± 1.24	54.5 ± 1.54	< 0.001^abc^	3 ± 0.003	24 ± 0.01	29 ± 0.01	< 0.001*
GREEN LEAFY VEGETABLES	10.5 ± 2.34	19.9 ± 2.28	34.9 ± 1.68	< 0.001^abc^	0.2 ± 0.001	2 ± 0.003	22 ± 0.01	< 0.001*
COOKING OIL	5.8 ± 0.32	4.7 ± 0.17	4.1 ± 0.16	< 0.001^ab^	17 ± 0.01	34 ± 0.01	21 ± 0.01	< 0.001*
OTHER CEREAL PRODUCTS	54.9 ± 1.37	54.9 ± 1.45	47.8 ± 2.51	< 0.001^bc^	48 ± 0.01	35 ± 0.01	20 ± 0.01	< 0.001*
OTHER VEGETABLES	27.9 ± 4.73	45.6 ± 3.11	55.4 ± 3.30	< 0.001^ab^	2 ± 0.003	10 ± 0.01	19 ± 0.01	< 0.001*
POWDERED MILK	15 ± 1.88	18.9 ± 0.88	16.5 ± 0.64	0.111	3 ± 0.003	21 ± 0.01	17 ± 0.01	< 0.001*
CHICKEN EGG	33.1 ± 1.25	40.5 ± 1.19	41.5 ± 1.65	< 0.001^ab^	8 ± 0.005	18 ± 0.01	13 ± 0.01	< 0.001*

^a^Values are mean ± standard error

^b^Values are percentage ± standard error

^†^Using Analysis of Covariance adjusted for age, sex, urbanity, wealth quintiles and energy intake at breakfast using Turkey HDS posthoc pairwise comparisons; subscripts represents significant difference between ^a^Tertile 1 vs Tertile 2, ^b^Tertile 1 vs Tertile 3, and ^c^Tertile 2 vs Tertile 3

^‡^Using Rank Biserial Correlation test; ^*^significant

### Usual energy and nutrient intakes at breakfast and NRF9.3 scores by age group

Table 6 presents the average NRF 9.3 scores and mean habitual intake of energy and nutrients at breakfast per age group. Children age 6–12 years has the highest NRF 9.3 average score with 417 which reflects to a healthier diet at breakfast compared to elderly with 347, adolescent (340) and also for adult (330) age groups which had the lowest scores. In all the age groups, breakfast contributed approximately 328 kcal to 440 kcal of their daily intake with the significant highest mean intakes of energy among adolescents (440 kcal) and adults (426 kcal). In terms of macronutrients, the highest mean intakes for total carbohydrates at breakfast were significantly observed among adults (77 g) followed by adolescents (77 g), elderly (64 g) and lastly, school-aged children (54 g). Mean protein intakes were also considerably highest in adolescents (13 g) and lowest in children (10 g). This similar trend is also observed with other nutrients such as fiber, niacin, vitamin D, magnesium, and potassium. With regards to total fat, saturated fat, monounsaturated fat (MUFA), polyunsaturated fat (PUFA), cholesterol, sodium, and iron, mean intakes were significantly highest among adolescents and lowest in the elderly. Highest mean intakes of total sugar and calcium were seen among the elderly while vitamin A intake was significantly higher among adults.

**Table 6:** Mean NRF9.3 score and usual energy and nutrients at breakfast

	**6–12 yo**	**13–18 yo**	**19–59 yo**	**60 yo + **	***P*-value**
	**(*n* = 13, 801)**	**(*n* = 8, 916)**	**(*n* = 31, 091)**	**(*n* = 7,989)**	
NRF 9.3. Score	417.5 ± 1.33	339.6 ± 2	330.1 ± 1.1	347.8 ± 2.2	< 0.001^abcef^
Energy (kcal)	328.4 ± 2	440 ± 3.3	426 ± 1.9	352.3 ± 2.8	< 0.001^abcef^
**Macronutrients**					
Total Protein (g)	10.2 ± 0.1	13.3 ± 0.1	12.9 ± 0.1	10.8 ± 0.1	< 0.001^abcef^
Total Carbohydrate (g)	54.4 ± 0.3	76.6 ± 0.6	76.9 ± 0.3	63.7 ± 0.5	< 0.001^abcdef^
Total Fat (g)	7.9 ± 0.1	9 ± 0.1	7.4 ± 0.1	6.1 ± 0.1	< 0.001^abcdef^
Saturated Fat (g)	3.2 ± 0.04	3.8 ± 0.1	3.1 ± 0.03	2.5 ± 0.05	< 0.001^acdef^
MUFA (g)	2.3 ± 0.04	2.5 ± 0.05	2 ± 0.02	2 ± 0.04	< 0.001^abcdef^
PUFA (g)	1.1 ± 0.01	1.2 ± 0.02	1.1 ± 0.01	1 ± 0.02	< 0.001^adef^
Total Fiber (g)	1.4 ± 0.01	1.9 ± 0.02	1.9 ± 0.01	1.7 ± 0.02	< 0.001^abcdef^
Total Sugar (g)	7.8 ± 0.1	8.6 ± 0.1	10.9 ± 0.1	11.2 ± 0.1	< 0.001^abcdef^
**Vitamins and Minerals**					
Thiamin (mg)	0.2 ± 0.003	0.22 ± 0.003	0.19 ± 0.002	0.16 ± 0.002	< 0.001^abcdef^
Riboflavin(mg)	0.22 ± 0.003	0.21 ± 0.003	0.2 ± 0.002	0.19 ± 0.003	< 0.001^abcdef^
Niacin (mg)	3 ± 0.03	4.2 ± 0.04	4.5 ± 0.02	3.8 ± 0.04	< 0.001^abcdef^
Vitamin C (mg)	3.3 ± 0.1	3.4 ± 0.1	3.5 ± 0.1	4 ± 0.2	< 0.001^bcdef^
Vitamin A	90.6 ± 2.7	84.4 ± 2.8	92.7 ± 3.5	76.5 ± 3.1	0.008^f^
Vitamin D	0.6 ± 0.01	0.7 ± 0.02	0.8 ± 0.01	0.7 ± 0.02	< 0.001^abcdf^
Calcium (mg)	84.5 ± 1	85.3 ± 1.3	80 ± 0.8	87.8 ± 1.6	< 0.001^cef^
Phosphorus (mg)	164.1 ± 1.2	208.5 ± 1.9	196.4 ± 1.1	169.5 ± 1.8	< 0.001^abcef^
Iron (mg)	2 ± 0.02	2.3 ± 0.03	2.1 ± 0.01	1.9 ± 0.02	< 0.001^abcdef^
Magnesium	28.8 ± 0.2	39.3 ± 0.4	38.8 ± 0.2	33.7 ± 0.4	< 0.001^abcdef^
Potassium	193.4 ± 1.6	271.1 ± 2.7	317.6 ± 1.6	284.2 ± 3.1	< 0.001^abcdef^
Sodium (mg)	266 ± 3.9	301.5 ± 5.7	261.1 ± 3.2	232.4 ± 5	< 0.001^acdef^
**Non-essential nutrient**					
Cholesterol	44.2 ± 0.7	47.8 ± 1.1	43.2 ± 0.5	34 ± 0.9	< 0.001^cdef^

The data shown were mean ± standard error weighted to the Philippine population

*P*-value showed significant difference between ^a^6-12yo VS 13–18 yo, ^b^6-12yo VS 19–59 yo, ^c^6-12yo VS 60yo + , ^d^13-18yo VS 19-59yo, ^e^13-18yo VS 60yo + , ^f^19-59yo VS 60yo +

### Usual energy and nutrient intakes at breakfast by NRF9.3 tertiles

Tables 7, 8, 9 and 10 showed the mean intake of usual energy and nutrients across NRF9.3 tertiles. In Table 7, results showed cumulative trend of energy intake at breakfast across NRF9.3 tertiles. There was a significant trend in all nutrients across NRF9.3 tertiles at breakfast among children except for total fat and saturated fat. Also, increasing consumption were observed across NRF9.3 tertiles for energy, protein, carbohydrates, fiber, thiamin, riboflavin, niacin, vitamin C, vitamin A, vitamin D, calcium, phosphorus, iron, magnesium, potassium, and cholesterol, while a decreasing trend was seen for MUFA, PUFA, total sugar and sodium intake. In Table 8, consumption of all nutrients among adolescents showed significant trends including energy intake. Moreover, consumption of total fat, saturated fat, MUFA, PUFA, total sugar and sodium declined across NFR tertiles while other nutrients significantly increased. For adults and elderly groups in Tables 9 and 10, both groups had the same results which was all nutrients including energy intake showed significant increasing trends across NRF tertiles. Total sugar and sodium intake decreases from tertile 1 to tertile 3. Results emphasize that the consumption of the population group in Tertile 3 consumed more essential nutrients such as vitamins and minerals and less intake of sodium, total sugar and total fat.

**Table 7:** Mean energy and nutrient intakes at breakfast of Children by NRF9.3 Score in Tertiles

	**All**	**Children aged 6- 12 years old (*n* = 13, 801)**			***P*-value**
		**T1**	**T2**	**T3**	
Energy (kcal)	328.4 ± 2	305.4 ± 3.8	362.8 ± 3.2	318.7 ± 3	< 0.001^**+**^
**Macronutrients**					
Total Protein (g)	10.2 ± 0.1	8 ± 0.1	11.8 ± 0.1	11 ± 0.1	< 0.001^**+**^
Total Carbohydrate (g)	54.4 ± 0.3	51 ± 0.6	59 ± 0.5	53.6 ± 0.5	< 0.001^**+**^
Total Fat (g)	7.9 ± 0.1	7.9 ± 0.2	8.8 ± 0.2	6.8 ± 0.1	0.991^NS^
Saturated Fat (g)	3.2 ± 0.04	3.2 ± 0.1	3.8 ± 0.1	2.7 ± 0.05	0953^NS^
MUFA (g)	2.3 ± 0.04	2.4 ± 0.1	2.6 ± 0.1	1.9 ± 0.04	0.003^−^
PUFA (g)	1.1 ± 0.01	1.1 ± 0.03	1.2 ± 0.02	0.9 ± 0.02	< 0.001^−^
Total Fiber (g)	1.4 ± 0.01	1.3 ± 0.02	1.4 ± 0.02	1.7 ± 0.02	< 0.001^**+**^
Total Sugar (g)	7.8 ± 0.1	9 ± 0.2	6.3 ± 0.2	8 ± 0.2	< 0.001^**−**^
**Vitamins and Minerals**					
Thiamin (mg)	0.2 ± 0.003	0.13 ± 0.003	0.15 ± 0.003	0.35 ± 0.01	< 0.001^**+**^
Riboflavin(mg)	0.22 ± 0.003	0.13 ± 0.002	0.22 ± 0.004	0.33 ± 0.01	< 0.001^**+**^
Niacin (mg)	3 ± 0.03	2.2 ± 0.03	3.1 ± 0.04	7.8 ± 0.2	< 0.001^**+**^
Vitamin C (mg)	3.3 ± 0.1	0.7 ± 0.1	1.7 ± 0.1	7.8 ± 0.2	< 0.001^**+**^
Vitamin A	90.6 ± 2.7	31.3 ± 1.2	79.7 ± 1.7	168.8 ± 8.1	< 0.001^**+**^
Vitamin D	0.6 ± 0.01	0.1 ± 0.01	0.6 ± 0.02	1.1 ± 0.03	< 0.001^**+**^
Calcium (mg)	84.5 ± 1	53.5 ± 1.3	88.1 ± 2.1	115.7 ± 1.8	< 0.001^**+**^
Phosphorus (mg)	164.1 ± 1.2	120.2 ± 1.9	185.7 ± 2	191.3 ± 2	< 0.001^**+**^
Iron (mg)	2 ± 0.02	1.5 ± 0.03	1.8 ± 0.03	2.7 ± 0.05	< 0.001^**+**^
Magnesium	28.8 ± 0.2	19 ± 0.3	29.5 ± 0.4	39.3 ± 0.5	< 0.001^**+**^
Potassium	193.4 ± 1.6	144.9 ± 2.4	200.4 ± 2.6	240.8 ± 3.2	< 0.001^**+**^
Sodium (mg)	266 ± 3.9	377.3 ± 8.2	240.6 ± 6.4	166.5 ± 4.1	< 0.001^**−**^
**Non-essential nutrient**					
Cholesterol	44.2 ± 0.7	15.6 ± 0.6	62 ± 1.3	58.2 ± 1.5	< 0.001^**+**^

The data shown were mean ± standard error weighted to the Philippine population

*NS* Not Significant

*P*-value showed significant ^+^increasing trend or ^−^decreasing trend

**Table 8:** Mean energy and nutrient intake from breakfast of Adolescents by NRF 9.3 Score in Tertiles

	**All**	**Adolescents aged 13–18 years old (*n* = 8,916)**			***P*-value**
		**T1**	**T2**	**T3**	
Energy (kcal)	440 ± 3.3	383.1 ± 5.7	498.4 ± 5.6	445.5 ± 5.4	< 0.001^**+**^
**Macronutrients**					
Total Protein (g)	13.3 ± 0.1	9.3 ± 0.2	15.2 ± 0.2	15.7 ± 0.2	< 0.001^**+**^
Total Carbohydrate (g)	76.6 ± 0.6	67.4 ± 1	87.2 ± 1.1	73.3 ± 0.9	< 0.001^**+**^
Total Fat (g)	9 ± 0.1	8.5 ± 0.2	9.7 ± 0.2	8.5 ± 0.2	< 0.001^−^
Saturated Fat (g)	3.8 ± 0.1	3.6 ± 0.1	4.2 ± 0.1	3.4 ± 0.1	< 0.001^−^
MUFA (g)	2.5 ± 0.05	2.3 ± 0.1	2.6 ± 0.1	2.4 ± 0.01	< 0.001^−^
PUFA (g)	1.2 ± 0.02	1.1 ± 0.03	1.2 ± 0.03	1.2 ± 0.03	< 0.001^−^
Total Fiber (g)	1.9 ± 0.02	1.5 ± 0.03	1.9 ± 0.03	2.4 ± 0.05	< 0.001^**+**^
Total Sugar (g)	8.6 ± 0.1	10.3 ± 0.2	6.7 ± 0.2	8.2 ± 0.2	< 0.001^−^
**Vitamins and Minerals**					
Thiamin (mg)	0.22 ± 0.003	0.15 ± 0.003	0.19 ± 0.004	0.33 ± 0.01	< 0.001^**+**^
Riboflavin(mg)	0.21 ± 0.003	0.12 ± 0.002	0.20 ± 0.003	0.32 ± 0.01	< 0.001^**+**^
Niacin (mg)	4.2 ± 0.04	2.9 ± 0.04	4.5 ± 0.1	5.3 ± 0.1	< 0.001^**+**^
Vitamin C (mg)	3.4 ± 0.1	0.5 ± 0.1	1.7 ± 0.1	7.3 ± 0.2	< 0.001^**+**^
Vitamin A	84.4 ± 2.8	17.5 ± 0.9	67.8 ± 1.6	142.2 ± 2.7	< 0.001^**+**^
Vitamin D	0.7 ± 0.02	0.1 ± 0.01	0.6 ± 0.02	1.5 ± 0.04	< 0.001^**+**^
Calcium (mg)	85.3 ± 1.3	42.7 ± 1	79.8 ± 1.6	130.9 ± 2.5	< 0.001^**+**^
Phosphorus (mg)	208.5 ± 1.9	138.3 ± 2.7	236 ± 2.7	256.7 ± 3.5	< 0.001^**+**^
Iron (mg)	2.3 ± 0.03	1.7 ± 0.04	2.2 ± 0.04	3 ± 0.05	< 0.001^**+**^
Magnesium	39.3 ± 0.4	24.2 ± 0.4	40.3 ± 0.5	53.6 ± 0.8	< 0.001^**+**^
Potassium	271.1 ± 2.7	192.6 ± 3	265.5 ± 3.5	357.7 ± 5.7	< 0.001^**+**^
Sodium (mg)	301.5 ± 5.7	400.8 ± 9.8	262.7 ± 8.3	200.5 ± 6.9	< 0.001^−^
**Non-essential nutrient**					
Cholesterol	47.8 ± 1.1	18.5 ± 1	68.1 ± 1.9	59.1 ± 2.2	< 0.001^**+**^

The data shown were mean ± standard error weighted to the Philippine population

*NS* Not Significant

*P*-value showed significant ^+^increasing trend or ^−^decreasing trend

**Table 9:** Mean energy and nutrient intake at breakfast of Adults by NRF 9.3 Score in Tertiles

	**All**	**Adults aged 19–59 years old (*n* = 31, 091)**			***P*-value**
		**T1**	**T2**	**T3**	
Energy (kcal)	426 ± 1.9	290.3 ± 2.6	514.8 ± 3.3	494.9 ± 0.1	< 0.001^+^
**Macronutrients**					
Total Protein (g)	12.9 ± 0.1	6.4 ± 0.1	15.2 ± 0.1	18.1 ± 0.1	< 0.001^+^
Total Carbohydrate (g)	76.9 ± 0.3	54.1 ± 0.4	92.5 ± 0.6	87.7 ± 0.5	< 0.001^+^
Total Fat (g)	7.4 ± 0.1	5.3 ± 0.1	9.1 ± 0.1	7.9 ± 0.1	< 0.001^+^
Saturated Fat (g)	3.1 ± 0.03	2.2 ± 0.05	4 ± 0.05	3.2 ± 0.04	< 0.001^+^
MUFA (g)	2 ± 0.02	1.4 ± 0.03	2.4 ± 0.04	2.2 ± 0.04	< 0.001^+^
PUFA (g)	1.1 ± 0.01	0.9 ± 0.01	1.2 ± 0.02	1.2 ± 0.01	< 0.001^+^
Total Fiber (g)	1.9 ± 0.01	1.1 ± 0.01	2 ± 0.02	2.8 ± 0.02	< 0.001^+^
Total Sugar (g)	10.9 ± 0.1	13.3 ± 0.1	9.4 ± 0.1	9.1 ± 0.1	< 0.001^−^
**Vitamins and Minerals**					
Thiamin (mg)	0.19 ± 0.002	0.10 ± 0.001	0.18 ± 0.002	0.28 ± 0.003	< 0.001^+^
Riboflavin(mg)	0.2 ± 0.002	0.11 ± 0.001	0.20 ± 0.002	0.28 ± 0.03	< 0.001^+^
Niacin (mg)	4.5 ± 0.02	2.6 ± 0.02	4.9 ± 0.04	6.2 ± 0.05	< 0.001^+^
Vitamin C (mg)	3.5 ± 0.1	0.2 ± 0.02	1.5 ± 0.05	7.8 ± 0.14	< 0.001^+^
Vitamin A	92.7 ± 3.5	12.5 ± 0.4	61.9 ± 0.8	130.8 ± 1.4	< 0.001^+^
Vitamin D	0.8 ± 0.01	0.1 ± 0.004	0.6 ± 0.01	1.6 ± 0.02	< 0.001^+^
Calcium (mg)	80 ± 0.8	32.8 ± 0.5	80.1 ± 0.9	127.6 ± 1.2	< 0.001^+^
Phosphorus (mg)	196.4 ± 1.1	89.2 ± 1.1	234.3 ± 1.6	281.2 ± 1.8	< 0.001^+^
Iron (mg)	2.1 ± 0.01	1.2 ± 0.02	2.2 ± 0.02	2.9 ± 0.02	< 0.001^+^
Magnesium	38.8 ± 0.2	16 ± 0.2	41.5 ± 0.3	61.1 ± 0.4	< 0.001^+^
Potassium	317.6 ± 1.6	206.1 ± 1.5	307.1 ± 2.2	450.7 ± 3.3	< 0.001^+^
Sodium (mg)	261.1 ± 3.2	297.5 ± 4.5	266.2 ± 4.6	183.7 ± 3.6	< 0.001^−^
**Non-essential nutrient**					
Cholesterol	43.2 ± 0.5	12.5 ± 0.4	62.1 ±	58.1 ± 1.1	< 0.001^+^

The data shown were mean ± standard error weighted to the Philippine population

*NS* Not Significant

*P*-value showed significant ^+^increasing trend or ^−^decreasing trend

**Table 10:** Mean energy and nutrient intakes at breakfast of Elderly by NRF 9.3 Score in

	**All**	**Elderly aged 60 years and above (*n* = 7,989)**			***P*-value**
		**T1**	**T2**	**T3**	
Energy (kcal)	352.3 ± 2.8	267 ± 4.2	413.7 ± 5	386.5 ± 4.6	< 0.001^+^
**Macronutrients**					
Total Protein (g)	10.8 ± 0.1	6 ± 0.1	12.7 ± 0.2	14.1 ± 0.2	< 0.001^+^
Total Carbohydrate (g)	63.7 ± 0.5	49.9 ± 0.7	73.9 ± 0.9	68.9 ± 0.8	< 0.001^+^
Total Fat (g)	6.1 ± 0.1	4.8 ± 0.2	7.4 ± 0.2	5.9 ± 0.2	< 0.001^+^
Saturated Fat (g)	2.5 ± 0.05	1.9 ± 0.1	3 ± 0.1	2.3 ± 0.1	< 0.001^+^
MUFA (g)	2 ± 0.04	1.2 ± 0.05	2 ± 0.1	1.7 ± 0.1	< 0.001^+^
PUFA (g)	1 ± 0.02	0.9 ± 0.03	1.2 ± 0.03	1 ± 0.02	< 0.001^+^
Total Fiber (g)	1.7 ± 0.02	1 ± 0.02	1.7 ± 0.03	2.6 ± 0.05	< 0.001^+^
Total Sugar (g)	11.2 ± 0.1	13.4 ± 0.2	10.1 ± 0.2	9.4 ± 0.2	< 0.001^−^
**Vitamins and Minerals**					
Thiamin (mg)	0.16 ± 0.002	0.1 ± 0.002	0.16 ± 0.003	0.22 ± 0.005	< 0.001^+^
Riboflavin(mg)	0.19 ± 0.003	0.1 ± 0.002	0.21 ± 0.004	0.24 ± 0.005	< 0.001^+^
Niacin (mg)	3.8 ± 0.04	2.5 ± 0.04	4.1 ± 0.1	4.8 ± 0.1	< 0.001^+^
Vitamin C (mg)	4 ± 0.2	0.2 ± 0.03	1.7 ± 0.1	9.1 ± 0.3	< 0.001^+^
Vitamin A	76.5 ± 3.1	15.2 ± 0.9	65.3 ± 1.7	122.5 ± 2.8	< 0.001^+^
Vitamin D	0.7 ± 0.02	0.1 ± 0.01	0.6 ± 0.02	1.3 ± 0.04	< 0.001^+^
Calcium (mg)	87.8 ± 1.6	35.9 ± 1	90.9 ± 2.2	133.6 ± 2.6	< 0.001^+^
Phosphorus (mg)	169.5 ± 1.8	81 ± 1.7	198.6 ± 2.6	236.4 ± 3.3	< 0.001^+^
Iron (mg)	1.9 ± 0.02	1.2 ± 0.03	1.9 ± 0.03	2.5 ± 0.05	< 0.001^+^
Magnesium	33.7 ± 0.4	15.2 ± 0.3	34.4 ± 0.5	52.2 ± 0.7	< 0.001^+^
Potassium	284.2 ± 3.1	192.8 ± 2.8	268.6 ± 3.8	394.2 ± 6.7	< 0.001^+^
Sodium (mg)	232.4 ± 5	264 ± 8	255.8 ± 8.3	159.6 ± 5.8	< 0.001^+^
**Non-essential nutrient**					
Cholesterol	34 ± 0.9	13.4 ± 0.9	47.3 ± 1.8	41.9 ± 1.7	< 0.001^+^

The data shown were mean ± standard error weighted to the Philippine population

*NS* Not Significant

*P*-value showed significant ^+^increasing trend or ^−^decreasing trend

### Contribution of energy and nutrients at breakfast to the total daily intake

**Fig. 2:**
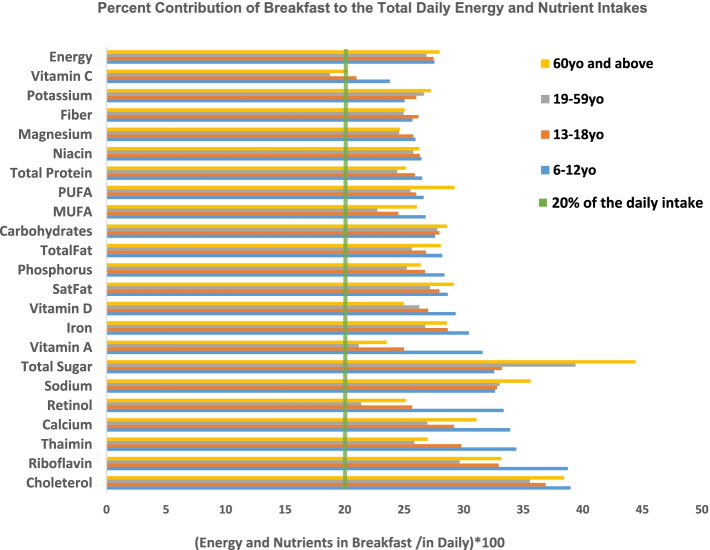
Energy and nutrient contribution of breakfast to the total daily intake. *PUFA stands for polyunsaturated fatty acids, MUFA stands for Monounsaturated fatty acids, SatFat stands for saturated fatty acids. The 20% cutoff is indicated by a vertical line with color green bar

Figure 2 shows the energy and nutrient contribution of breakfast to the total daily intake. Breakfast must consist at least 20–25% of the total daily energy and nutrients. Overall, energy and nutrients coming from breakfast reached more than the 20% of the total daily intake of energy and nutrients. On the other hand, total sugar, cholesterol, and sodium contributed to more than 30% at breakfast for all age groups. Total sugar accounted for 40% of daily consumption at breakfast for adults aged 19–49 years old and goes up to approximately 45% for the elderly. Contribution of vitamin C at breakfast seems to be low especially for adults aged 19–59 years old which means that vitamin C intake was low during breakfast.

### Contribution of breakfast to the daily recommended intakes

**Fig. 3:**
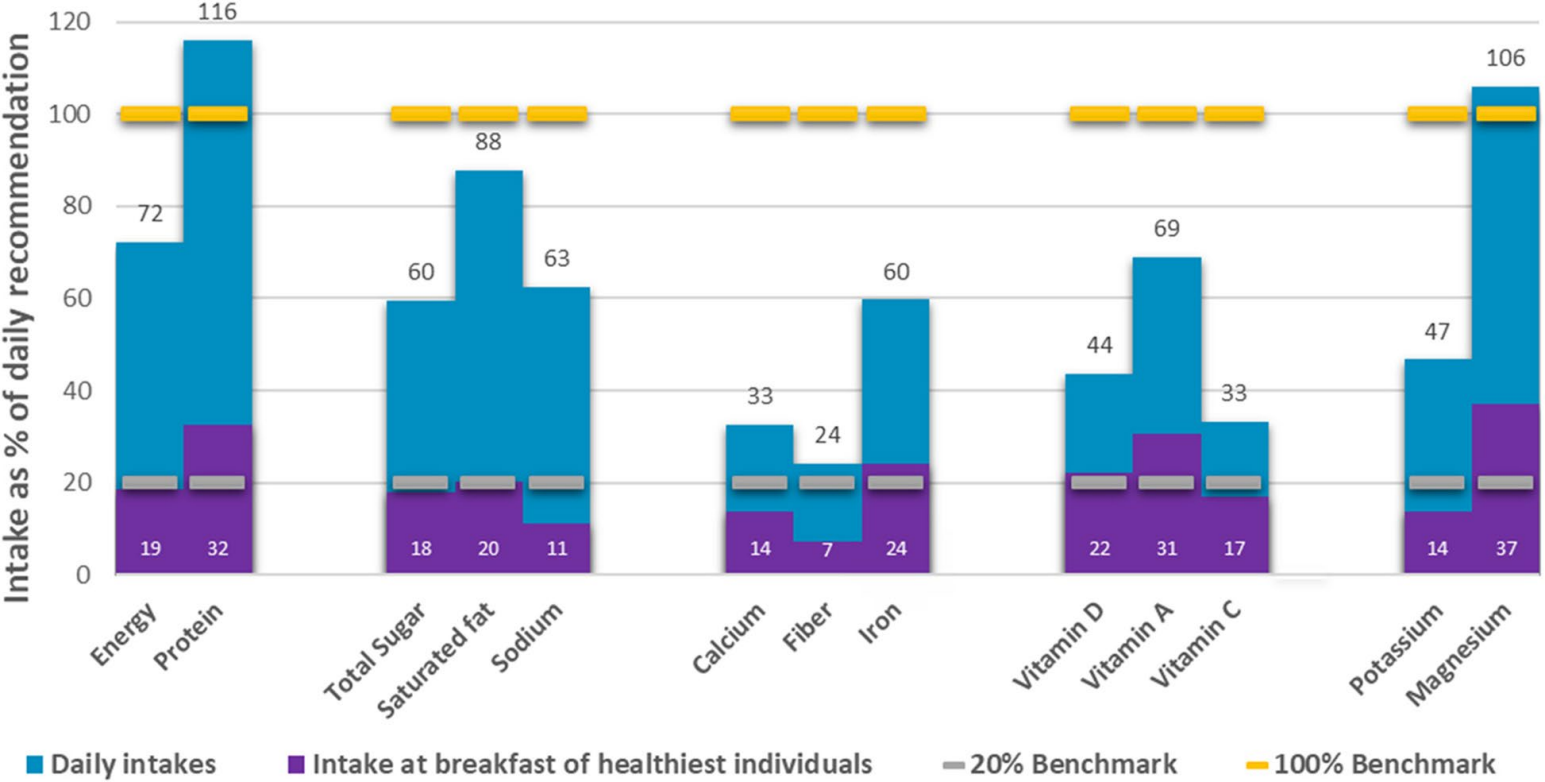
Contribution of breakfast to the daily recommendation among children NOTE: Daily Intake = All meals; Intake at breakfast of healthiest individuals = intake from breakfast only of participants that were categorized in Tertile 3 which represent the individual with healthiest diet; 20% Benchmark = 20% of the recommended intake of each nutrient (based on the PDRI), 100% Benchmark = the total recommendation per day

Figure 3 showed that children with the healthiest breakfast diets met the 20% recommendation for intake of protein (32%), iron (24%), vitamin D (22%), vitamin A (31%) and magnesium (37%) but fall short in energy (19%), calcium (14%), fiber (7%), vitamin C (17%) and potassium (14%) intakes at breakfast. On a daily basis, intake of magnesium and protein were more than adequate but there were deficiencies in calcium, fiber, iron, vitamin D, vitamin C, and potassium. There were no recorded excessive intakes of total sugar, saturated fat, and sodium intake at breakfast, same was observed in the results for daily consumption. Results also showed that there is a low nutrient consumption at breakfast.

**Fig. 4:**
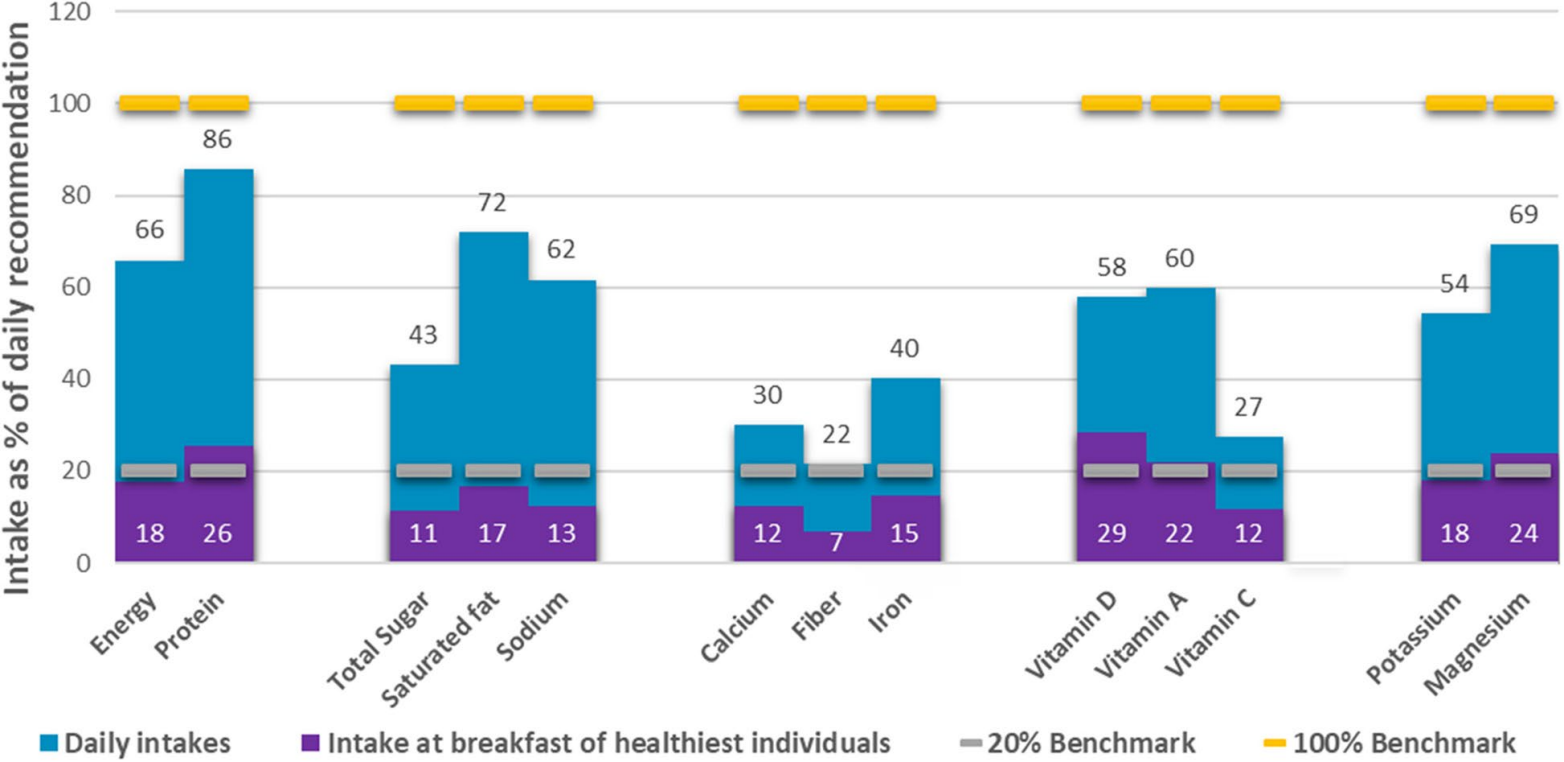
Contribution of breakfast to the daily recommendation among adolescent. NOTE: Daily Intake = 24 h food recall (All meals); Intake at breakfast of healthiest individuals = intake from breakfast only of participants that were categorized in Tertile 3 which represent the individual with healthiest diet; 20% Benchmark = 20% of the recommended intake of each nutrient (based on the PDRI), 100% Benchmark = the total recommendation per day

Adolescents with the healthiest breakfast diets met the 20% recommended intake of protein (26%), vitamin D (29%), vitamin A (22%) and magnesium (24%). Only 7% of fiber at breakfast was consumed. Poor intakes of calcium (12%), iron (15%), and vitamin C (12%) were observed at breakfast for adolescents. There is a slightly low contribution (11%) of total sugar, saturated fat (17%), and sodium (13%) for this group. Overall, it is noticeable that most micronutrients were inadequate especially calcium, fiber and vitamin C (Fig. 4).

**Fig. 5:**
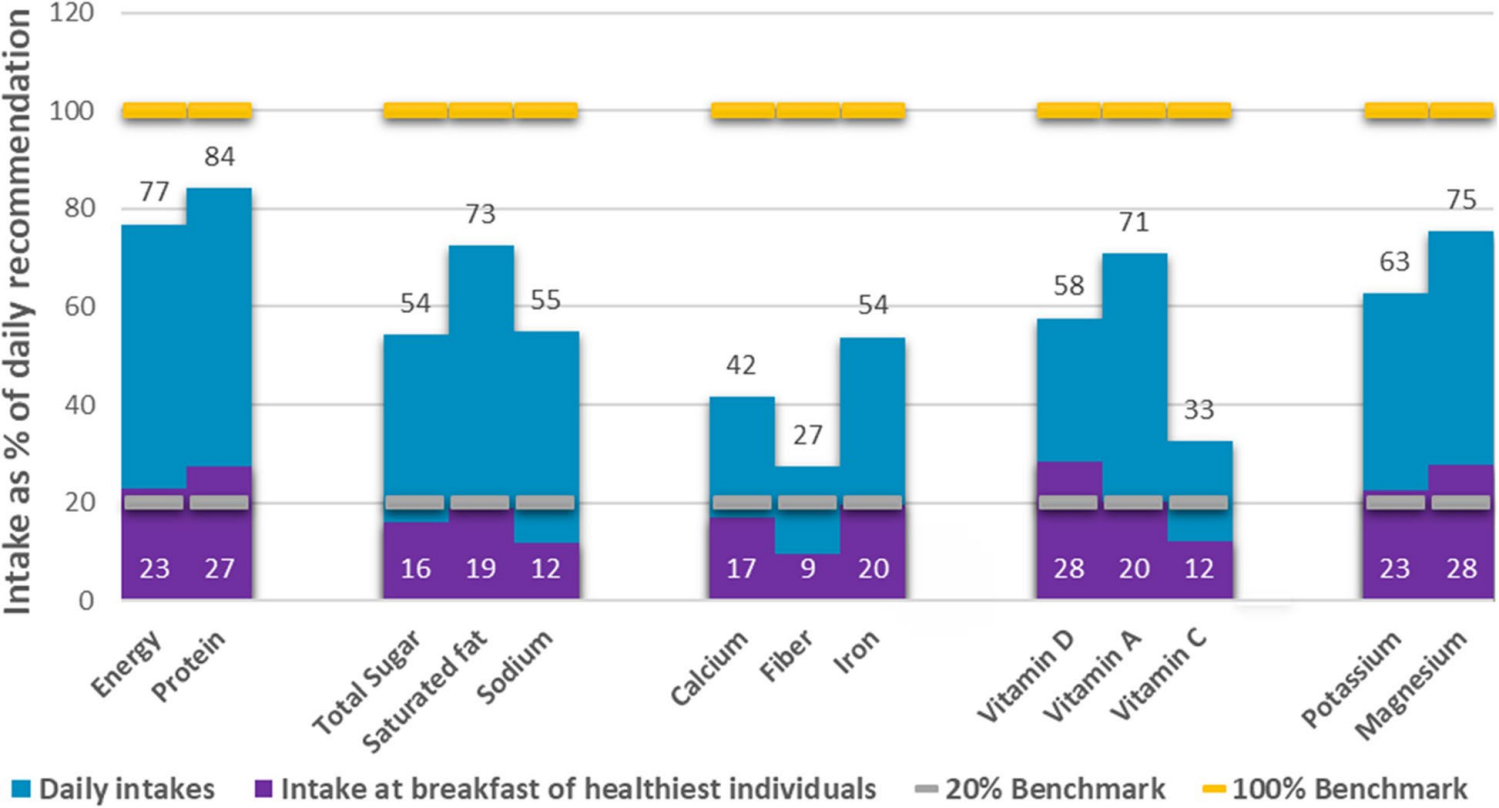
Contribution of breakfast to the daily recommendation among adults. NOTE: Daily Intake = 24 h food recall (All meals); Intake at breakfast of healthiest individuals = intake from breakfast only of participants that were categorized in Tertile 3 which represent the individual with healthiest diet; 20% Benchmark = 20% of the recommended intake of each nutrient (based on the PDRI), 100% Benchmark = the total recommendation per day

Adults with the healthiest breakfast diets (Tertile 3) were adequate in energy (23%), protein (27%), iron (20%), vitamin D (28%), vitamin A (20%), potassium (23%) and magnesium (28%) at breakfast. However, contribution of calcium (17%), vitamin C (12%), and fiber (9%) at breakfast is low, which is an unceasing problem up until this age group (Fig. 5).

**Fig. 6:**
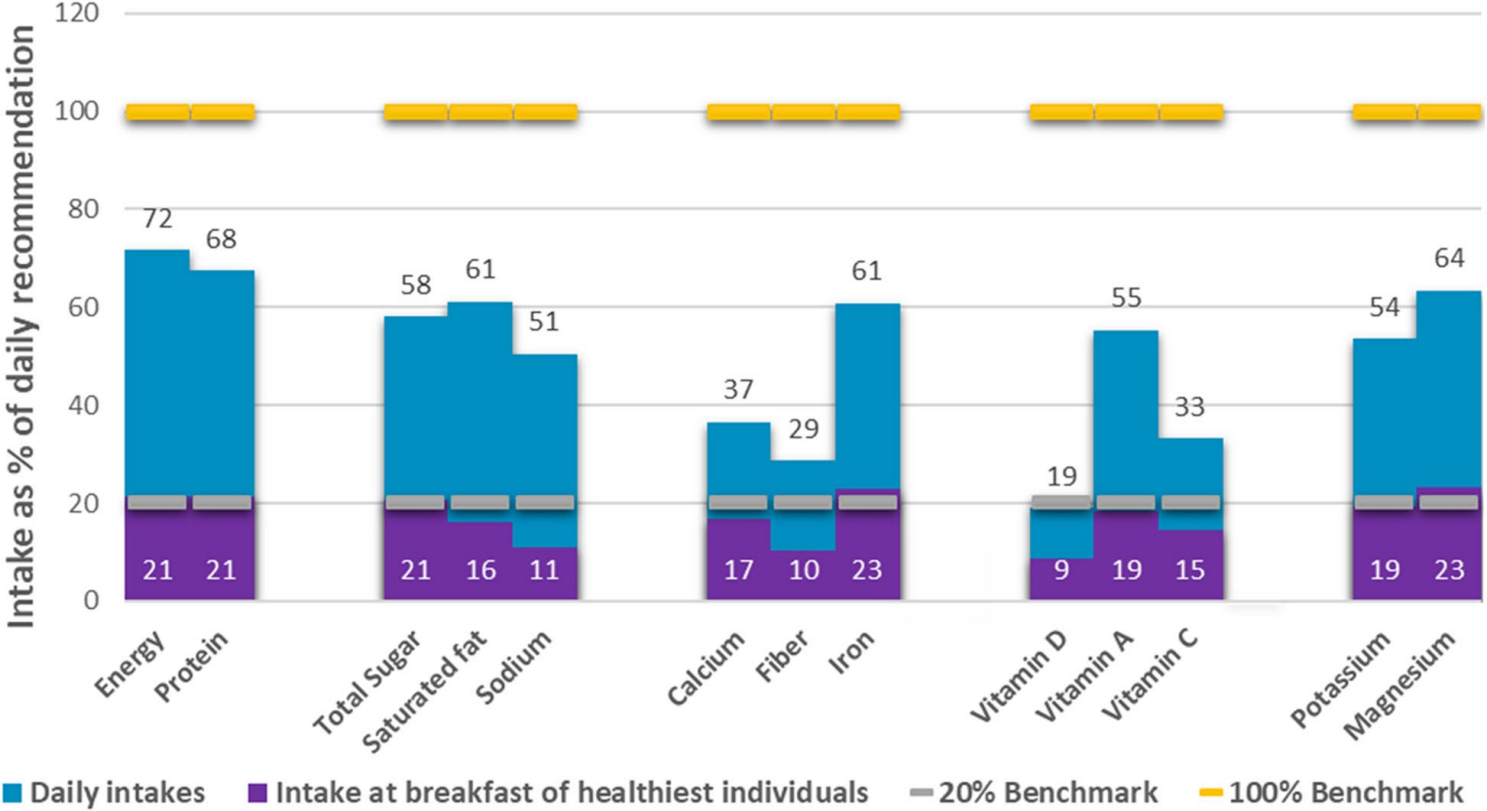
Contribution of breakfast to the daily recommendation among elderly. NOTE: Daily Intake = 24 h food recall (All meals); Intake at breakfast of healthiest individuals = intake from breakfast only of participants that were categorized in Tertile 3 which represent the individual with healthiest diet; 20% Benchmark = 20% of the recommended intake of each nutrient (based on the PDRI), 100% Benchmark = the total recommendation per day

Among the elderly with the healthiest breakfast diets, their breakfast consumption reached more than the 20% recommended intake of energy (21%), protein (21%), iron (23%), and magnesium (23%). However, the composition of their breakfast was inadequate in fiber (10%), vitamin D (9%), vitamin C (15%), and calcium (17%). There was only 1% excessiveness of total sugar (21%), and no excessiveness of saturated fat and sodium. On a daily basis, they were inadequate of calcium, fiber, vitamin D, and vitamin C (Fig. 6).

## Discussion

The present study determined the regularity of breakfast consumption and its contribution to the daily energy and nutrient intakes of Filipinos in order to serve as basis for breakfast recommendations in the Philippines. To our knowledge, the present study was probably the first attempting to describe the breakfast consumption of Filipinos. With the use of the secondary data from the 2018 ENNS gathered by the DOST-FNRI, this study was able to analyze the breakfast intakes of Filipinos [21]. Although the initial objective of the IBRI consortium was to pull together breakfast studies in Southeast Asia to come up with a prevailing unified recommendation for the region, this has been proven a challenge because of the varied breakfast definitions across countries, the different meal occasions, and the variety of food groups consumed at breakfast. Hence, the breakfast profile of Filipinos remained as the immediate topic of investigation in this study.

### Breakfast regularity

Results from the analysis reported that majority of the Filipino population (96-98%) regularly consume breakfast. This strongly identifies that breakfast is an important meal for Filipinos. As stated in a previous literature, the regularity of breakfast consumption and meal times were closely related to healthy lifestyle habits and could play an important role in providing adequate nutrients [31–33].

### Commonly consumed food and nutrient intakes at breakfast in relation to diet quality

In terms of breakfast energy and nutrient intakes, the present analyses also allowed this study to identify food choices and breakfast patterns that were associated with the highest quality of breakfast diets, as captured by NRF 9.3 scores. Participants were divided into tertiles based on their NRF 9.3 scores for each age group to investigate the associations between breakfast nutrient intake and overall diet quality. The age groups with the highest NRF 9.3 score or Tertile 3 (group with the healthiest breakfast) were characterized in this study to consume higher amounts of essential nutrients and more diversified diets as per food groups consumption, compared to lower tertiles. Moreover, a higher NRF score was associated with a higher consumption of desirable food groups making the index an ideal measurement of overall diet quality.

Upon analysis, the current study reported that energy intakes at breakfast does differ across NRF 9.3 tertiles. More so, it was revealed that Filipino children aged 6–12 years had the highest NRF average scores (417), followed by elderly (347), adolescents (340), and adults (330). Yet, all these scores were still considered to be very low in comparison with the maximum possible NRF score which is 900; and if compared with the findings from previous IBRI studies conducted in Western countries [7, 10]. These results show that majority of the Filipinos regularly consume breakfast, yet the breakfasts they were consuming reflected a meal of poor diet quality as per NFR 9.3 scores.

Filipino children and adolescents with the healthiest breakfast diets (Tertile 3) were associated with higher intakes of energy, total protein, total carbohydrates, total fiber, and micronutrients (except total sugar and sodium) compared to counterparts (Tertile 1 and Tertile 2). While for Filipino adults and elderly, those consuming the healthiest quality of breakfast diet exhibited higher intakes of energy, total protein, total carbohydrates, total fat, MUFA, PUFA, total fiber, micronutrients (except total sugar and sodium), and cholesterol. Even so, participants classified under the group with the healthiest diet (Tertile 3) still did not meet 20% of the dietary recommendations for calcium, fiber, vitamin C, and potassium, which clearly represents an opportunity for needed improvement.

These results were reflected in the breakfast consumption of food groups as higher intakes of fresh fish, chicken egg, fresh meat, green leafy vegetables and other vegetables, and lower intakes of rice, coffee, sugars, and other cereal products were found among groups with the healthiest breakfast diet. These food groups are included in the 2012 Nutritional Guidelines for Filipinos (NGF) which reflects the impact of its consumption towards a healthier diet [34]. The current study suggests that healthier food patterns at breakfast could be further examined in order to identify key food patterns in relation to nutrient intakes [35]. It was also found that the composition of breakfast among the healthiest individuals were similar to the recommended food groups to be consumed as stated in the Pinggang Pinoy Guidelines [36].

The investigation for the main sources of these aforementioned nutrients that Filipinos were found to be inadequate had revealed that the food groups that were associated as the richest sources of these nutrients were not consumed at breakfast. According to the food consumption survey, vitamin C intakes in the Philippines had an 83%-95% inadequacy rate which is very alarming [10]. This is reflected on the low levels of fruit and vegetable consumption at breakfast among Filipinos which are abundant sources of vitamin C, dietary fiber, and potassium []. Moreover, the survey also reported that most of the calcium intake came from rice (17–23%) which may not provide rich amounts of calcium as compared to milk and dairy products [38]. Previous literatures have pointed that low dietary intake of calcium and fiber may be a significant risk factor for obesity, thus increasing the risk for those who have inadequate calcium and fiber intakes [39,40]. Ingestion of a certain amount of dietary fiber apart from alleviating constipation also reduces hunger, thereby reducing total energy intake and preventing weight gain [40]. Additionally, prior research stated that low vitamin C intakes may put people at risk of developing clinical scurvy, which is fatal if left untreated [41] and low potassium intakes increase the risk of people for hypertension and other cardiovascular diseases [42]. Lower sodium and sugar intakes were also observed among Filipinos with the healthiest diet quality (tertile 3). Findings from previous literatures reported the impact of dietary sodium and sugar intake to cardiovascular disease risk, which recommends that lower sodium and sugar intakes should be encouraged to prevent the onset of heart diseases [43].

### Contribution of breakfast to daily intakes

Among Filipino breakfast consumers, more than 20% of the total daily energy intake was from breakfast. This finding is in line with a previous breakfast study conducted in Denmark [35], which indicates a relatively staple contribution of breakfast to the total energy intake. Moreover, the definition of nutrient density in the 2005 Dietary Guidelines defined nutrient-dense foods as those containing “more nutrients than calories” [45]. Thus, based on a simple nutrients-to-energy ratio, breakfast can be considered as a nutrient-rich meal. In contrast, breakfast contributed more than 30% of the daily intakes for total sugar, cholesterol, and sodium, which were higher than the contribution of energy and other micronutrients to the daily diet. It was also worth to note that vitamin C was poorly consumed during breakfast which is alarming, since breakfast meals have a huge impact to one’s daily nutrient intake. A vast majority of previous studies showed a clear overall nutritional benefit of consuming breakfast in respect of the key nutrients of public health importance in many countries [7, 10, 35, 46].

Indeed, results in this study had revealed that emphasis should be given on the consumption of fruits, vegetables, milk, and fresh meat which were among the least consumed food groups during breakfast among the Filipino population. Yet, it is also alarming that fruits as well as milk and milk products were found further down the list of mostly consumed food groups among Filipinos. When considered together, the poor food choices at breakfast are likely contributing to the suboptimal intake of some nutrients at this meal (e.g. high contribution of added sugar, and low contribution to vitamin C, fiber, potassium intakes) [], which could be improved by the higher consumption of fruits, vegetables, fresh fish, fresh meat, and milk, and lower consumption of refined grains, coffee and sugary products.

## Limitations of the study

Datasets for the food recalls did not record whether the food recalls were on a weekday or weekend. With the large sample size used in this study, this consideration could already be factored-in. The usage of dietary supplements was not included in this study since the goal was to estimate nutrient intakes through food and beverages only. This might result in an underestimate of total daily micronutrient consumption.

## Conclusion

This study revealed that majority of the population are regular breakfast consumers. However, the breakfast consumed by Filipinos were found to be of poor quality. And even those classified under tertile 3 which were assumed as having a better quality of breakfast were still found to have nutrient inadequacies. Based on the findings obtained, it could be suggested that Filipinos must consume a healthy breakfast by including nutrient-dense foods such as fruits, vegetables, whole grains, fresh meat, and milk to provide at least 20–25% of the daily energy and nutrient intakes. The results from this study also provide strong evidence for a positive impact of breakfast consumption on overall diet quality. These findings could help to inform the development of nutrient-based recommendations for a balanced breakfast for the first time in the Philippines.

The findings of this study opened a window of opportunity in improving the diet quality of breakfast in the Philippines by re-emphasizing recommendations in the Pinggang Pinoy and on the consumption of nutrient-dense foods. A future favorable turnout of the study would be the expansion of the national feeding program with the inclusion of breakfast and the possibility of extending the feeding until the adolescent age. As per Republic Act 11,037, which is the signed law that enacts free meals at public schools for undernourished kids, the current national nutrition program partnered by the Department of Education (DepEd) and Department of Social Welfare and Development (DSWD) only provides lunch meals to preschool until elementary children. Expansion from breakfast to lunch and targeting up until the adolescent age may help in mitigating micronutrient deficiencies through age groups, since breakfast is consumed by majority of the population.

## Supplementary Information

**Additional file 1:** **Table S1.** Age- and sex- specific reference daily values for young age group used for the calculation of NRF 9.3. **Table S2.** Age- and sex- specific reference daily values for adult age group used for the calculation of NRF 9.3.

**Additional file 2:** **Table S3.** Food Group Classification.

## Data Availability

Publicly available data from the 2018 Expanded National Nutrition Survey by the Food and Nutrition Research Institute – Department of Science and Technology is available at http://enutrition.fnri.dost.gov.ph/. Raw data sets are available on request from the corresponding author.

## Abbreviations

NNS: National Nutrition
NRF: Nutrient Rich Food
IBRI: International Breakfast Research Initiative
DOST – FNRI: Department of Science and Technology – Food and Nutrition Research Institute
PSA: Philippines Statistics Authority
PSU: Primary Sampling Unit
IDES: Individual Dietary Evaluation System
FCT: Food Composition Table
INFOODS: International Network of Food Data System
DRV: Daily Reference Value
RDI: Recommended Dietary Intake
PDRI: Philippines Recommended Dietary Intake
IOM: Institute of Medicine
NCEP: National Cholesterol Education Program
ANOVA: Analysis of Variance
ANCOVA: Analysis of Covariance
CED: Chronic Energy Deficiency
NAMD: Nutritional Assessment and Monitoring Division
