# Supplementary material for: Breakfast in the Philippines: food and diet quality as analyzed from the 2018 Expanded National Nutrition Survey
Source: Nutr J. 2022 Aug 12;21:52. doi: 10.1186/s12937-022-00804-x (PMC9373515; doi:10.1186/s12937-022-00804-x)
Supplement: Supplementary file 1 — Additional file 1: Table S1. Age- and sex- specific reference daily values for young age group used for the calculation of NRF 9.3. Table S2. Age- and sex- specific reference daily values for adult age group used for the calculation of NRF 9.3. [file 12937_2022_804_MOESM1_ESM.docx]

**Table S1**. Age- and sex- specific reference daily values for **young age group** used for the calculation of NRF 9.3

|  | 6 – 9 years old | | 10 – 12 years old | | 13 – 15 years old | | 16 – 18 years old | |
| --- | --- | --- | --- | --- | --- | --- | --- | --- |
|  | Male | Female | Male | Female | Male | Female | Male | Female |
| Energy (kcal) ^a^ | 1600 | 1470 | 2060 | 1980 | 2700 | 2170 | 3010 | 2280 |
| Energy (kJ) ^a^ | 6694 | 6151 | 8619 | 8284 | 11297 | 9079 | 12594 | 9540 |
| *Nutrients to Encourage* | | | | | | | | |
| Protein (g)^b^ | 30 | 29 | 43 | 46 | 62 | 57 | 73 | 61 |
| Dietary fibre (g)^c^ | 22.4 | 20.6 | 28.8 | 27.7 | 37.8 | 30.4 | 42.1 | 31.9 |
| Vitamin A (μg RAE) ^b^ | 400 | 400 | 500 | 500 | 700 | 500 | 800 | 600 |
| Vitamin C (mg) ^b^ | 45 | 45 | 45 | 45 | 60 | 55 | 70 | 60 |
| Vitamin D (µg)^b^ | 5 | 5 | 5 | 5 | 5 | 5 | 5 | 5 |
| Calcium (mg) ^b^ | 700 | 700 | 1000 | 1000 | 1000 | 1000 | 1000 | 1000 |
| Iron (mg) ^b^ | 10 | 9 | 12 | 20 | 19 | 28 | 14 | 28 |
| Potassium (mg) ^d^ | 1600 | 1600 | 2000 | 2000 | 2000 | 2000 | 2000 | 2000 |
| Magnesium (mg) ^b^ | 90 | 90 | 150 | 160 | 220 | 210 | 265 | 230 |
| *Nutrients to Limit* | | | | | | | | |
| Total sugars (g)^e^ | 40.0 | 36.75 | 51.5 | 49.5 | 67.5 | 54.3 | 75.3 | 57.0 |
| Saturated Fats (g)^f,g^ | 12.4 | 11.4 | 16.0 | 15.4 | 21.0 | 16.9 | 23.4 | 17.7 |
| Sodium (mg)^h^ | 1200 | 1200 | 1500 | 1500 | 1500 | 1500 | 1500 | 1500 |

^Dietary Reference Values were derived from the 2015 Philippine Dietary Reference Intakes a. Recommended Energy Intakes per day; b. Recommended Nutrient Intakes per day; c. 14 g fiber per 1,000 kcal = basis for AI for fiber. d. Adequate Intake Levels, PDRI 2015; e. WHO Guideline on Sugar Intake for Adults and Children (2015): free sugars refer to all monosaccharides and disaccharides added to foods and drinks by the manufacturer, cook or consumer, including sugars naturally present in honey, syrups, fruit juices and fruit concentrates, (<10% of energy); f. Therapeutic Lifestyle Changes (TLC) recommendations. g. Determined based on the Tentative Dietary Goals for Japanese people (7% of energy); h. Based on the Adequate Intake Recommendations of the Institute of Medicine (IOM)^ ^Dietary reference intakes for water, potassium, sodium, chloride, and sulfate, 2004;^

**Table S2**. Age- and sex- specific reference daily values for **adult age group** used for the calculation of NRF 9.3

|  | 19 – 29 years old | | 30 - 49 years old | | 50 - 59 years old | | 60 - 69 years old | | ≥70 years old | |
| --- | --- | --- | --- | --- | --- | --- | --- | --- | --- | --- |
|  | Male | Female | Male | Female | Male | Female | Male | Female | Male | Female |
| Energy (kcal) ^a^ | 2530 | 1930 | 2420 | 1870 | 2420 | 1870 | 2140 | 1610 | 1960 | 1540 |
| Energy (kJ) ^a^ | 10586 | 8075 | 10125 | 7824 | 10125 | 7824 | 8954 | 6736 | 8201 | 6443 |
| *Nutrients to Encourage* | | | | | | | | | | |
| Protein (g)^b^ | 71 | 62 | 71 | 62 | 71 | 62 | 71 | 62 | 71 | 62 |
| Dietary Fibre (g)^c^ | 35.4 | 27.0 | 33.9 | 26.2 | 33.9 | 26.2 | 30.0 | 22.5 | 27.4 | 21.6 |
| Vitamin A (μg RAE)^b^ | 700 | 600 | 700 | 600 | 700 | 600 | 700 | 600 | 700 | 600 |
| Vitamin C (mg) ^b^ | 70 | 60 | 70 | 60 | 70 | 60 | 70 | 60 | 70 | 60 |
| Vitamin D (µg) ^i^ | 5 | 5 | 5 | 5 | 10 | 10 | 15 | 15 | 15 | 15 |
| Calcium (mg) ^b^ | 750 | 750 | 750 | 750 | 750 | 800 | 800 | 800 | 800 | 800 |
| Iron (mg) ^b^ | 12 | 28 | 12 | 28 | 12 | 10 | 12 | 10 | 12 | 10 |
| Potassium (mg) ^d^ | 2000 | 2000 | 2000 | 2000 | 2000 | 2000 | 2000 | 2000 | 2000 | 2000 |
| Magnesium (mg) ^i^ | 240 | 210 | 240 | 210 | 240 | 210 | 240 | 210 | 240 | 210 |
| *Nutrients to Limit* | | | | | | | | | | |
| Total sugars (g)^e^ | 63.3 | 48.3 | 60.5 | 46.8 | 60.5 | 46.8 | 53.5 | 40.3 | 49.0 | 38.5 |
| Saturated Fats (g)^f,g^ | 19.7 | 15.0 | 18.8 | 14.5 | 18.8 | 14.5 | 16.6 | 12.5 | 15.2 | 12.0 |
| Sodium (mg)^h^ | 1500 | 1500 | 1500 | 1500 | 1300 | 1300 | 1300 | 1300 | 1200 | 1200 |

^Dietary Reference Values were derived from the 2015 Philippine Dietary Reference Intakes a. Recommended Energy Intakes per day; b. Recommended Nutrient Intakes per day; c. 14 g fiber per 1,000 kcal = basis for AI for fiber. d. Adequate Intake Levels, PDRI 2015; e. WHO Guideline on Sugar Intake for Adults and Children (2015): free sugars refer to all monosaccharides and disaccharides added to foods and drinks by the manufacturer, cook or consumer, including sugars naturally present in honey, syrups, fruit juices and fruit concentrates, (<10% of energy); f. Therapeutic Lifestyle Changes (TLC) recommendations. g. Determined based on the Tentative Dietary Goals for Japanese people (7% of energy); h. Based on the Adequate Intake Recommendations of the Institute of Medicine (IOM) Dietary reference intakes for water, potassium, sodium, chloride, and sulfate, 2004; i. Recommended Nutrient Intakes.^

***Assessment of Dietary Values***

Dietary reference values (DRV) were determined based on the Philippine Dietary Reference Intake 2015 which presents the available recommendations for the 9 recommended nutrients and 3 limiting nutrients that will be used in the computation of the NRF9.3. The PDRI 2015 is an age- and sex- specific reference on the daily nutrient values to meet the needs of various stakeholders for the appropriate nutrient reference values. More information on this reference can be viewed at the corresponding reference (1).

Consistent with the initial IBRI, the emerging public health deficiency on vitamin D has been a cause of concern for different countries especially since several researches have conclusive on the protective effect of vitamin D is an against various diseases such as osteoporosis, colon cancer and several viral pathogens, the initial inclusion of vitamin E to the 9 recommended nutrients in the computation of the NRF9.3 has been replaced by vitamin D (2), (3), (4).

Sodium recommendations included in the PDRI are based on the WHO Guideline on Sodium Intake for Adults and Children (2012) which states to limit the intake of sodium to less than 2g in adults (5). To enable to derive a specific dietary reference value which is also age and gender specific, recommendations from the Institute of Medicine (IOM) Dietary Reference Intakes for Tolerable Upper Intake Levels of sodium based on age and gender (6).

With regards to added sugar, there is currently no specific recommendation adapted for the Filipino population in the Philippine Dietary Reference Intakes rather it considered the recommendation for free sugar intake published by the World Health Organization in 2015 (7). This recommendation was further evaluated with the actual intakes of the Philippine population [ (8), (9) thus it was concluded to use the *conditional recommendation* (upper limit in the <5% of energy) WHO since there is low intake of the nutrient in the Filipino population and to lessen risks and disadvantages when it comes to nutrient recommendations that has low totality evidence reviewed with the chosen populace.

Another nutrient that is missing in the PDRI is saturated fat. The recommendation that is included in Tables S1 and S2 are based on the recommendation by the National Cholesterol Education Program (NCEP) specifically for Therapeutic Lifestyle Change of <7% of energy from saturated fat (10). This is also in conjunct to the Tentative Dietary Goals by the Japanese population (11). The values are adjusted to meet the age- and gender- specific reference values consistent with the other nutrients.

References:

1. **Department of Science and Technology - Food and Nutrition Research Institute.** the Philippine Dietary Reference Intake 2015. [Online] 2015. https://www.fnri.dost.gov.ph/index.php/159-fnri-launches-the-philippine-dietary-reference-intakes-pdri-2015.

2. *Vitamin D Status and Usual Nutrient Intake of Filipino Children Aged 6-12 Years in Selected Areas in the Philippines: A 2018 National Nutrition Survey.* **Angeles-Agdeppa, Imelda and Tanda, Keith.** 2021, Journal of Nutrition and Metabolism, pp. 1-9.

3. *Vitamin D and anemia: insights into an emerging association.* **Smith, Ellen M and Tangpricha, Vin.** 6, s.l. : Current Opinion in Endocrinology,Diabetes and Obesity, 2015, Vol. 22, pp. 432-8.

4. *Vitamin D-deficiency in Asia.* **Fraser, David R.** 1-5, s.l. : the Journal of Steriod Biochemistry and Molecular Biology, 2004, Vols. 89-90, pp. 491-5.

5. **World Health Organization.** Sodium Intake for Adults and Children. [Online] https://www.who.int/elena/titles/guidance_summaries/sodium_intake/en/#:~:text=WHO%20recommends%20a%20reduction%20to%20%3C2%20g/day%20sodium,requirements%20of%20children%20relative%20to%20those%20of%20adults..

6. **The National Academies of Sciences Engineering Medicine.** Dietary Reference Intakes for Sodium and Potassium. [Online] 2019. https://www.nap.edu/catalog/25353/dietary-reference-intakes-for-sodium-and-potassium.

7. **World Health Organization.** Guideline: sugars intake for adults and children. [Online] March 4, 2015. https://www.who.int/publications/i/item/9789241549028.

8. *Food sources, energy and nutrient intakes of adults: 2013 Philippines National Nutrition Survey.* **Angeles-Agdeppa, Imelda, et al.** 1, s.l. : Nutrition Journal, October 2019, Vol. 18.

9. *Dietary pattern and nutrient intakes in association with non-communicable disease risk factors among Filipino adults: a cross-sectional study.* **Angeles-Agdeppa, Imelda, Sun, Ye and Tanda, Keith V.** 1, s.l. : Nutrition Journal, 2020, Vol. 19.

10. **National Cholesterol Education Program.** National Cholesterol Education Program (NCEP) Guidelines for Interpretation of Lipid Values. [Online] 2004. https://www.pubinfo.vcu.edu/pathLabs/print%20menu/appendix_ncep_guidelines.pdf.

11. **the Japan Dietetic Association.** Overview of Dietary Reference Intakes for Japanese. [Online] 2015. https://www.mhlw.go.jp/file/06-Seisakujouhou-10900000-Kenkoukyoku/Overview.pdf.
