# Supplementary material for: Breakfast in the Philippines: food and diet quality as analyzed from the 2018 Expanded National Nutrition Survey
Source: Nutr J. 2022 Aug 12;21:52. doi: 10.1186/s12937-022-00804-x (PMC9373515; doi:10.1186/s12937-022-00804-x)
Supplement: Supplementary file 2 — Additional file 2: Table S3. Food Group Classification. [file 12937_2022_804_MOESM2_ESM.docx]

| **Table S3. Food Group Classification** | | | |
| --- | --- | --- | --- |
|  | **Food Groups** | **Sub-group** | **Food Items** |
| 1 | **CORN** | *Milled* | corn grits, cornmeal |
|  |  | *Corn On The Cob* | corn on cob |
|  |  | *Others* | baby corn, canned corn, hominy |
| 2 | **CORN PRODUCTS** | *Cornstarch* | cornstarch, corn chips, pop corn |
| 3 | **OTHER CEREAL PRODUCTS** | *Bread (As Flour)* | cheese roll, monay, toasted, loaf, doughnut |
|  |  | *Cakes/Pastries (As Flour)* | cheesecakes, cupcake, pie, pudding |
|  |  | *Cookies/Biscuits (As Flour)* | cookies, biscuits |
|  |  | *Flour (In Own Form)* | hard wheat, all-purpose flour |
|  |  | *Noodles* | instant noodles, pasta |
|  |  | *Other Cereal Products* | cereals, chips, curls, pizza, hamburger |
|  |  | *Pandesal (As Flour)* | bread, pandesal |
| 4 | **RICE** | *Glutinous* | glutinous |
|  |  | *Ordinary* | well-milled |
|  |  | *Special* | undermilled, glutinous, purple |
| 5 | **RICE PRODUCTS** | *Rice Noodles* | rice gruels |
|  |  | *Rice Cakes* | rice cake |
| 6 | **MUNGBEANS AND PRODUCTS** | *Mung beans And Products* | mongo |
| 7 | **NUTS AND PRODUCTS** | *Peanuts* | peanut |
| 8 | **OTHER DRIED BEANS SEEDS PRODUCT** | *Other Dried Beans/Seeds And Product* | chickpea, kidney beans, |
| 9 | **SOYBEANS AND PRODUCTS** | *Other Soybean Products* | soybean cheese, soybean pudding |
|  |  | *Soybean Milk Preparations* | soy bean drinks |
|  |  | *Soybeans* | seed dried |
|  |  | *Soy sauce* | soy sauce |
| 10 | **DUCK EGG** | *Duck's Eggs* | duck egg |
| 11 | **CHICKEN EGG** | *Chicken's Eggs* | chicken egg |
| 12 | **OTHER EGG** | *Other Eggs* | ant, quail, turtle |
| 13 | **BUTTERFAT** | *Butter (As Fat)* | butter |
| 14 | **COCONUT** | *Coconut Cream (Gata)* | coconut milk, coconut milk powdered |
|  |  | *Coconut Grated (As Fat)* | coconut meat, mature |
| 15 | **COOKING OIL** | *Cooking Oil (Vegetable)* | coconut, palm, corn, soybean, sunflower oil |
| 16 | **MARGARINE** | *Margarine* |  |
| 17 | **OTHER FATS AND OILS** | *Other Fats And Oils* | mayonnaise, coconut cream curd |
| 18 | **PEANUT BUTTER** | *Peanut Butter* | peanut butter |
| 19 | **PORK DRIPPINGS AND LARD** | *Pork Drippings And Lard* | beef tallow, pork fat |
| 20 | **CRUSTACEANS AND MOLLUSKS** | *Bagoong (As Fresh)* | fish paste, shrimp paste |
|  |  | *Crabs* | crab |
|  |  | *Dried And Processed (As Fresh)* | canned squid, dried shrimp, dried squid |
|  |  | *Others (Fresh)* | clam, oyster, sea cucumber |
|  |  | *Shrimp (All Types)* | shrimp |
|  |  | *Squid/Octopus* | squid |
|  |  | *Mussels* | mussels, green |
| 21 | **DRIED FISH** | *Dried Fish (As Fresh Fish)* | anchovy, catfish, cod, mudfish, tilapia, tuna |
| 22 | **FERSH FISH** | *Albakora/Tambakol* | tambakol |
|  |  | *Alumahan* | alumahan |
|  |  | *Balila/Espada* | barla |
|  |  | *Bangus* | bangus |
|  |  | *Bisugo* | bisugo |
|  |  | *Dalagang Bukid* | dalagang bukid |
|  |  | *Dilis Buo* | dilis |
|  |  | *Galunggong* | galunggong |
|  |  | *Hasa-Hasa* | hasa-hasa |
|  |  | *Lapu-Lapu* | lapu-lapu |
|  |  | *Matangbaka* | matang baka |
|  |  | *Maya-Maya* | isda |
|  |  | *Other Fresh Fish & Ckd. Fish Rec.* | buwan buwan |
|  |  | *Salay-Salay* | pikay |
|  |  | *Sapsap* |  |
|  |  | *Silinyasi/Tunsoy* | taubak |
|  |  | *Tamban* | tamban |
|  |  | *Tanigi* | tanigue |
|  |  | *Tilapya* | tilapia |
|  |  | *Tulingan* | tulingan |
| 23 | **FRESH MEAT** | *Beef* | beef |
|  |  | *Carabeef* | carabeef |
|  |  | *Other Fresh Meat* | goat meat |
|  |  | *Pork* | pork |
| 24 | **ORGAN MEAT** | *Carabao* | blood, intestine, liver |
|  |  | *Chicken And Other Poultry* | blood, gizzard, heart, intestine, liver |
|  |  | *Cow* | blood, heart, intestine, liver |
|  |  | *Liver Spread* | liver spread |
|  |  | *Organ Meat Recipes* | pork crackling, internal organ |
|  |  | *Other Organ Meats (Fresh)* | beef tongue, chevon |
|  |  | *Pig* | blood, heart, intestine, liver |
| 25 | **POULTRY** | *Chicken* | back, breast, feet, head , leg, wing, thigh |
|  |  | *Other Fowls* | duck breast, quail meat, duck thigh |
| 26 | **PROCESSED FISH** | *Bagoong (As Fresh Fish)* | fish paste |
|  |  | *Canned Fish* | *sardines, mackerel, etc.* |
|  |  | *Patis* | fish sauce |
|  |  | *Smoked Fish* | smoked fish |
| 27 | **PROCESSED MEAT** | *Canned Meat (As Fresh Meat)* | Chicken w/ veg, embotido |
|  |  | *Cooked Meat Recipes* | roasted chicken, pork back, pork belly, spring roll |
|  |  | *Popular Processed Meat* | bacon, beef jerky, meat balls, corn beef, sausages, burger patty |
| 28 | **CARROT AND OTHER YELLOW VEGETABLES** | *Carrot And Other Yellow Vegetables* | carrots, squash flower, |
| 29 | **GREEN LEAFY VEGETABLES** | *Alugbati* | malabar nightshade leaves |
|  |  | *Gabi Leaves* | taro leaves |
|  |  | *Kangkong* | swamp cabbage leaves |
|  |  | *Malunggay* | horseradish tree leaves |
|  |  | *Other Green Leafy And Cooked Vegs.* | bitter melon leaves, cassava leaves, celery, chayote, lettuce, |
|  |  | *Pechay* | Chinese cabbage, petchay leaves |
|  |  | *Sweet Potato Tops* | sweet potato leaves |
| 30 | **SQUASH FRUIT** | *Squash Fruit* | squash fruit |
| 31 | **CONDENSED MILK** | *Condensed Milk (As Whole Milk)* | milk, sweetened, condensed, filled |
| 32 | **EVAPORATED MILK** | *Evaporated Milk, Filled, Recomb, & Whole Milk* | milk, evaporated |
| 33 | **FRESH WHOLE MILK** | *Fresh Whole Milk* | cow’s milk. goat’s milk, |
| 34 | **MILK PRODUCTS** | *Cheese* | cheese spread, cheese, |
|  |  | *Other Milk Products* | cream cheese, cream, ice cream, yogurt, milk drink |
| 35 | **POWDERED MILK** | *Filled* | milk, powder, filled, instant |
|  |  | *Infant Formula* | infant formula milk |
|  |  | *Skimmed* | skimmed milk |
|  |  | *Whole/Full Cream* | full cream milk |
| 36 | **ALCOHOLIC BEVERAGES** | *Alcoholic Beverages* | beer, brandy, gin, rhum |
| 37 | **CACAO AND CHOCOLATE BASED BEVERAGES** | *Cacao And Chocolate-Based Beverage* | choco-flavored drink, chocolate beverage, cocoa |
| 38 | **COFFEE** | *Coffee* | pure instant coffee, 3in1 coffee, rice/corn/soybean coffee |
| 39 | **CONDIMENTS AND SPICES** | *Condiments And Spices* | catsup, curry powder, pepper, seasoning mixed |
| 40 | **OTHER BEVERAGES** | *Other Beverages* | energy drink, fruit juice drink, iced tea, milk tea, |
| 41 | **OTHER MISCELLANEOUS** | *Others* | artificial sweetener, breading mixed, coconut water, rice bran, yeast |
| 42 | **SALT** | *Salt* | course, iodized |
| 43 | **SOUPS** | *Soups* | cream of mushroom soup, Knorr cream soup |
| 44 | **TUBA** | *Tuba* | fermented palm sap |
| 45 | **VINEGAR** | *Vinegar* | cane, coconut, apple cider, palm, pineapple |
| 46 | **CANNED AND PROCESSED FRUITS AND VEGETABLES** | *Canned And Processed Vegetables* | canned mushroom, pickles, bamboo shoot, tomato sauce |
|  |  | *Fruit Juices* | Juice of mango, cranberry raspberry, pineapple, grapes |
|  |  | *Other Canned And Processed Fruits* | Halo-halo, jam, canned pineapple chunks |
| 47 | **OTHER FRUITS** | *All Other Fruits* | apple, avocado, dragon fruit, grapes, lanzones, java apple, pear, santol |
|  |  | *Bananas* | lacatan, latundan, murado, saba, wrapped fried saba with sugar |
|  |  | *Jackfruit* | jackfruit |
|  |  | *Kaimito* | star apple, purple |
|  |  | *Melon* | melon |
|  |  | *Pineapple* | pineapple |
|  |  | *Watermelon* | watermelon |
|  |  | *Young Coconut* | coconut meat |
| 48 | **OTHER VEGETABLES** | *Abitsuelas* | snap bean pod |
|  |  | *All Other Vegetables* | banana stem, broccoli, cauliflower, cucumber, mushroom, seaweeds |
|  |  | *Ampalaya (Bittergourd) Fruit* | bitter melon/gourd fruit |
|  |  | *Bamboo Shoot* | bamboo shoot |
|  |  | *Banana Heart* | banana heart |
|  |  | *Cabbage* | cabbage |
|  |  | *Eggplant* | eggplant |
|  |  | *Gabi Stalk* | taro petioles |
|  |  | *Garlic* | garlic bulb |
|  |  | *Ginger* | ginger |
|  |  | *Gourds (Bottle And Sponge)* | bottle gourd fruit, sponge gourd fruit |
|  |  | *Green Papaya* | papaya fruit |
|  |  | *Jackfruit (Unripe)* | jackfruit fruit |
|  |  | *Okra* | lady’s finger |
|  |  | *Onion* | onion bulb |
|  |  | *Other Fresh Leguminous Pods* | mung bean seed, snow/sugar pea pod, winged bean pod |
|  |  | *Pepper (All Variety)* | pepper |
|  |  | *Sayote (Fruit)* | Chayote fruit |
|  |  | *String beans* | String/Yard long bean pod, String/Yard long bean seed |
| 49 | **CASSAVA AND PRODUCTS** | *Cassava And Products* | Cassava, Cassava cake, Cassava suman |
| 50 | **OTHER STARCHY ROOTS AND TUBERS** |  | East Indian arrowroot, Palauan, yam |
| 51 | **POTATOES AND PRODUCTS** | *Potatoes And Products* | potato, Potato chips, Potato, french-fried |
| 52 | **SWEET POTATOES TUBERS AND PRODUCT** | *Sweet Potatoes, Tubers And Product* | Sweet potato |
| 53 | **JAMS AND OTHER SWEETS** | *Jams And Other Sweets* | candy bar, choco-coated mallow, coco jam, chocolate spread, marshmallow, syrup |
| 54 | **SHERBET ICE DROP ICE CANDY ETC** | *Sherbet, Icedrop, Ice Candy, Etc* | ice candy, ice drop, popsicle |
| 55 | **SOFTDRINKS** | *Softdrinks (Sugar Content)* | softdrinks |
| 56 | **SUGARS** | *Brown* | brown sugar |
|  |  | *Crude* | crude sugar, muscovado, molasses |
|  |  | *Refined* | refined white sugar |
| 57 | **CITRUS** | *Citrus Fruits* | calamansi, lemon fruit, orange, pomelo |
| 58 | **MANGO** | *Mango (Green, Semi-Ripe And Ripe)* | mango |
| 59 | **OTHER VITC RICH FOODS** |  | cashew fruit, durian, guava, rambutan, strawberry, |
| 60 | **PAPAYA** | *Papaya (Semi-Ripe And Ripe)* | papaya fruit |
| 61 | **TOMATOES** | *Tomatoes* | tomato |
